# Umbrella review of mesenchymal stem cell-derived extracellular vesicles in preclinical models: therapeutic efficacy across diverse conditions

**DOI:** 10.3389/fcell.2025.1655623

**Published:** 2025-10-13

**Authors:** Nadiar M. Mussin, Kulyash R. Zhilisbayeva, Akmaral Baspakova, Madina A. Kurmanalina, Amin Tamadon

**Affiliations:** ^1^ Department of General Surgery No. 2, West Kazakhstan Marat Ospanov Medical University, Aktobe, Kazakhstan; ^2^ Department of Languages, West Kazakhstan Marat Ospanov Medical University, Aktobe, Kazakhstan; ^3^ Department of Epidemiology, West Kazakhstan Marat Ospanov Medical University, Aktobe, Kazakhstan; ^4^ Department of Dental Disciplines and Maxillofacial Surgery, West Kazakhstan Marat Ospanov Medical University, Aktobe, Kazakhstan; ^5^ Department of Natural Sciences, West Kazakhstan Marat Ospanov Medical University, Aktobe, Kazakhstan

**Keywords:** mesenchymal stem cells, extracellular vesicles, exosomes, preclinical models, umbrella review, regenerative medicine

## Abstract

**Background:**

Mesenchymal stem cell-derived extracellular vesicles (MSC-EVs) have emerged as a promising cell-free therapeutic strategy for various diseases due to their anti-inflammatory, anti-apoptotic, and regenerative properties. Numerous meta-analyses have evaluated MSC-EV efficacy in preclinical animal models, but a comprehensive synthesis across diverse conditions is lacking.

**Objective:**

This umbrella review aims to systematically evaluate the therapeutic efficacy, mechanisms, and methodological quality of MSC-EVs in preclinical models across multiple diseases.

**Methods:**

A systematic search of Scopus and Web of Science was conducted to identify meta-analyses published up to July 2025, focusing on MSC-EV interventions in preclinical animal models. Data were extracted on study characteristics, exosome sources, animal models, outcomes, and risk of bias. The AMSTAR 2 tool assessed meta-analysis quality, while SYRCLE and CAMARADES tools evaluated primary study bias. Narrative and quantitative syntheses summarized efficacy, heterogeneity, and publication bias.

**Results:**

Forty-seven meta-analyses covering 27 diseases were included, spanning neurological, renal, wound healing, liver, musculoskeletal, respiratory, and reproductive disorders. MSC-EVs demonstrated high efficacy, significantly improving functional scores, reducing inflammation, and promoting regeneration. Bone marrow-, adipose-, and umbilical cord-derived EVs were most effective, with modified EVs showing enhanced outcomes. Methodological quality was moderate (AMSTAR 2), with high heterogeneity (I^2^ > 70%) and frequent risk of bias due to poor randomization and blinding. Publication bias was noted but often robust after adjustments.

**Conclusion:**

MSC-EVs exhibit robust therapeutic potential across diverse preclinical models, supporting their development as a versatile regenerative therapy. Standardization of EV protocols, improved study quality, and mechanistic insights are critical for clinical translation. This review provides a comprehensive framework for advancing MSC-EV research and application.

## 1 Introduction

Mesenchymal stem cells (MSCs) have garnered significant attention in regenerative medicine due to their multipotent differentiation capacity, immunomodulatory properties, and ability to promote tissue repair (Song et al., 2020). Derived from various sources such as bone marrow, adipose tissue, and umbilical cord, MSCs have shown therapeutic promise in preclinical and clinical studies across a wide range of conditions, including neurological, cardiovascular, renal, and musculoskeletal disorders (Zhidu et al., 2024). However, challenges such as immune rejection, variable efficacy, and potential tumorigenicity (Zhou et al., 2021) have prompted exploration of cell-free alternatives, particularly MSC-derived extracellular vesicles (MSC-EVs).

MSC-EVs, including exosomes and microvesicles, are nano-sized membrane-bound structures that carry bioactive molecules such as microRNAs, proteins, and lipids (Dabrowska et al., 2020). These vesicles mediate intercellular communication and recapitulate many of the therapeutic effects of their parent cells, including anti-inflammatory, anti-apoptotic, and regenerative actions (Kou et al., 2022). Unlike whole-cell therapies, MSC-EVs offer advantages such as lower immunogenicity, enhanced stability, and the ability to cross biological barriers, making them a promising platform for next-generation therapeutics (Kou et al., 2022). Preclinical studies in animal models have demonstrated MSC-EV efficacy in diverse conditions, from ischemic stroke (Zhao et al., 2023) and spinal cord injury (SCI) (Yi and Wang, 2021) to diabetic wounds (Soltani et al., 2024) and liver fibrosis (Zhou et al., 2024), highlighting their broad therapeutic potential.

Despite this promise, the field faces challenges, including variability in EV sources, isolation methods, and dosing regimens, as well as inconsistencies in preclinical study design and reporting (Dai et al., 2025). Numerous meta-analyses have synthesized evidence on MSC-EV efficacy for specific diseases, but a comprehensive overview integrating these findings across conditions is lacking. Umbrella reviews, which systematically synthesize meta-analyses, provide a high-level perspective to assess the consistency, quality, and generalizability of evidence, guiding future research and clinical translation.

This umbrella review aims to evaluate the therapeutic efficacy of MSC-EVs in preclinical animal models across diverse diseases. By analyzing outcomes, exosome sources, mechanisms of action, and methodological quality, we seek to provide a robust synthesis of the current evidence, identify gaps, and propose directions for advancing MSC-EV-based therapies. This work addresses the critical need for a unified understanding of MSC-EV potential, paving the way for standardized protocols and clinical applications.

## 2 Materials and methods

This umbrella review was conducted to systematically synthesize evidence from meta-analyses evaluating the therapeutic efficacy of MSC-EVs in preclinical animal models across diverse diseases and conditions. The methodology followed established guidelines for systematic reviews, including the Preferred Reporting Items for Systematic Reviews and Meta-Analyses (PRISMA) and the Joanna Briggs Institute (JBI) framework for umbrella reviews. Below, we detail the materials and methods used, organized into subsections for clarity.

### 2.1 Study design

This study is an umbrella review, defined as a systematic review of systematic reviews and meta-analyses. The objective was to aggregate and evaluate the therapeutic potential, mechanisms, and methodological quality of MSC-EV interventions in preclinical animal models. The review focused on meta-analyses to provide a high-level synthesis of evidence, capturing a broad range of diseases, exosome sources, and outcomes. The protocol was developed *a priori* and registered with PROSPERO to ensure transparency and reproducibility.

### 2.2 Search strategy

**FIGURE 1:**
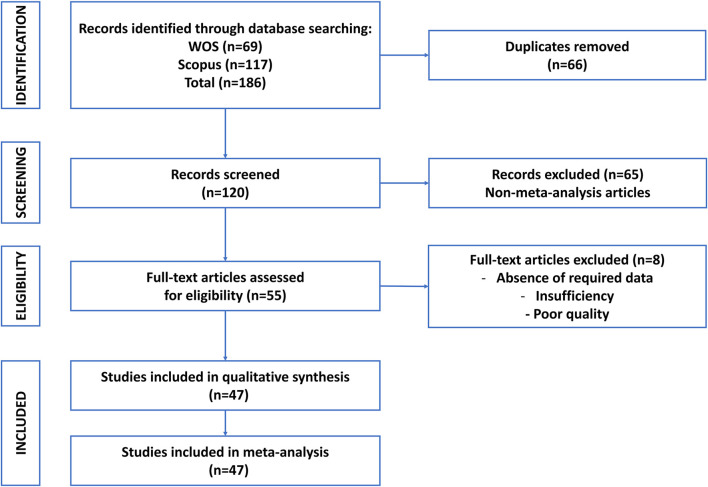
Flowchart of literature search and screening process for umbrella review of meta-analysis articles of mesenchymal stem cell-derived extracellular vesicles in preclinical models.

A comprehensive and systematic literature search was conducted to identify relevant meta-analyses. The search strategy was designed to capture studies evaluating MSC-EV therapeutic efficacy in preclinical models, with specific queries tailored to extracellular vesicles, mesenchymal stem cells, and meta-analyses (Table 1). The search was executed across multiple electronic databases, and the strategy was adapted from Table 1 of the provided article. The Scopus and Web of Science databases were searched from inception to July 2025 by two independent reviewers (N.M.M. and K.R.Z.) using standardized search protocols. Search results were exported to EndNote 20 for deduplication, and duplicates were removed using both automated and manual checks (Figure 1). The search strategy was validated by a medical librarian to ensure comprehensiveness and accuracy.

**TABLE 1:** Systematic search strategy for screening of meta-analysis articles evaluating mesenchymal stromal/stem cells-derived extracellular vesicles.

Code	Queries
#1	“Extracellular Vesicles” OR “Exosomes” OR “Extracellular Vesicle” OR “Vesicle, Extracellular” OR “Vesicles, Extracellular” OR “Exovesicles” OR “Exovesicle”
#2	“Mesenchymal Stem Cells” OR “Stem Cell, Mesenchymal” OR “Mesenchymal Stem Cell” OR “Stem Cells, Mesenchymal” OR “Mesenchymal Stromal Cells” OR “Mesenchymal Stromal Cell” OR “Stromal Cell, Mesenchymal” OR “Stromal Cells, Mesenchymal” OR “Wharton Jelly Cells” OR “Wharton’s Jelly Cells” OR “Wharton’s Jelly Cell” OR “Whartons Jelly Cells” OR “Bone Marrow Stromal Cells” OR “Bone Marrow Stromal Cell” OR “Bone Marrow Stromal Cells, Multipotent” OR “Multipotent Bone Marrow Stromal Cell” OR “Multipotent Bone Marrow Stromal Cells” OR “Bone Marrow Stromal Stem Cells” OR “Mesenchymal Progenitor Cell” OR “Mesenchymal Progenitor Cells” OR “Progenitor Cell, Mesenchymal” OR “Progenitor Cells, Mesenchymal” OR “Multipotent Mesenchymal Stromal Cells” OR “Mesenchymal Stromal Cells, Multipotent” OR “Multipotent Mesenchymal Stromal Cell” OR “Bone Marrow Mesenchymal Stem Cells” OR “Bone Marrow Mesenchymal Stem Cell” OR “Adipose-Derived Mesenchymal Stem Cells” OR “Adipose Derived Mesenchymal Stem Cells” OR “Adipose-Derived Mesenchymal Stromal Cells” OR “Adipose Derived Mesenchymal Stromal Cells” OR “Mesenchymal Stem Cells, Adipose-Derived” OR “Mesenchymal Stem Cells, Adipose Derived” OR “Adipose Tissue-Derived Mesenchymal Stromal Cell” OR “Adipose Tissue Derived Mesenchymal Stromal Cell” OR “Adipose Tissue-Derived Mesenchymal Stromal Cells” OR “Adipose Tissue Derived Mesenchymal Stromal Cells” OR “Adipose Tissue-Derived Mesenchymal Stem Cell” OR “Adipose Tissue Derived Mesenchymal Stem Cell” OR “Adipose Tissue-Derived Mesenchymal Stem Cells” OR “Adipose Tissue Derived Mesenchymal Stem Cells” OR “Adipose-Derived Mesenchymal Stem Cell” OR “Adipose Derived Mesenchymal Stem Cell”
#3	“meta-analysis” or “meta analysis”
#4	#1 AND #2 AND #3 (Filter: language restriction (English), Date limitation: up to 31 July 2025)

### 2.3 Eligibility criteria

For inclusion in this umbrella review, studies were selected based on predefined inclusion and exclusion criteria to ensure both relevance and methodological quality. Eligible studies were systematic reviews that included meta-analyses of preclinical studies, specifically those investigating MSC-EVs—including exosomes, microvesicles, or other EV subtypes—as the primary therapeutic intervention. Studies combining MSC-EVs with other therapies, such as scaffolds or pharmacological agents, were included provided that MSC-EVs remained the central focus. The target population comprised preclinical animal models used to study a broad range of diseases or conditions. Included studies had to report quantitative outcomes relevant to therapeutic efficacy, such as functional assessments, histological evaluations, molecular biomarkers, or survival rates. Only English-language, peer-reviewed journal articles were considered.

Studies were excluded if they were narrative reviews, systematic reviews without meta-analyses, or primary research articles. Additional exclusion criteria included studies that focused on EVs not derived from MSCs, unless MSC-EVs constituted a major component of the analysis. Clinical trials or studies involving human subjects were excluded, as were meta-analyses limited solely to *in vitro* data. Non-English publications, conference abstracts, grey literature, preprints, and other non-peer-reviewed materials were also excluded from this review.

### 2.4 Study selection

The study selection process was conducted in two distinct stages to ensure methodological rigor and transparency. In the first stage, titles and abstracts were independently screened by two reviewers (A.B. and M.A.K.). This initial screening was performed against the predefined eligibility criteria. Any discrepancies between the reviewers were resolved through discussion or, if necessary, by consulting a third reviewer (A.T.). In the second stage, the full texts of studies deemed potentially eligible were retrieved and independently evaluated by two additional reviewers (A.B. and M.A.K.) to determine their final inclusion. At this stage, specific reasons for exclusion were carefully documented. To provide a clear overview of the selection process, a PRISMA flow diagram was generated, outlining the number of records identified, screened, included, and excluded at each phase of the review (Figure 1).

### 2.5 Data extraction

Data extraction was carried out independently by two reviewers (N.M.M. and K.R.Z.) using a standardized form developed in Microsoft Excel. This form was piloted on five studies to ensure consistency, clarity, and completeness in data capture. After extraction, data were cross-verified for accuracy by the reviewers. Any inconsistencies were resolved through consensus or, when necessary, by consulting a senior author (A.T.).

The data extraction encompassed several key elements. For study characteristics, information was collected on the authors, year of publication, journal name, and reference number, along with the total number of studies included in each meta-analysis and the specific disease or condition being investigated. Intervention details included the type of MSC-EVs, the origin of the MSCs, and the method of delivery.

Regarding animal models, data were gathered on the species used, the specific strains, and the experimental disease models employed. Outcomes extracted included both primary outcomes and secondary outcomes. Where available, effect sizes such as standardized mean differences (SMD), weighted mean differences (WMD), hazard ratios (HR), or odds ratios (OR) were recorded, along with their corresponding 95% confidence intervals. Measures of heterogeneity, such as the I^2^ statistic, were also documented.

In terms of methodological quality, each study’s risk of bias was assessed using established tools like SYRCLE or CAMARADES. The overall risk of bias was categorized as low, moderate, high, or unclear. Evaluation of publication bias included methods such as Egger’s test and visual inspection of funnel plots. Furthermore, the AMSTAR 2 tool was used to appraise the methodological quality of the included systematic reviews and meta-analyses, with ratings categorized as high, moderate, low, or critically low, and critical flaws explicitly noted. Data were extracted from main texts, tables, and Supplementary Material. When numerical data was missing, attempts were made to contact the original authors for clarification. In cases where no response was obtained, data were estimated from graphical figures.

### 2.6 Quality assessment

To evaluate the methodological rigor of the included meta-analyses and the risk of bias in the primary studies they synthesized, two complementary assessment tools were employed. The AMSTAR 2 was used to appraise the overall quality of the included meta-analyses. Two independent reviewers (A.B. and G.A.T.) applied the 16-item checklist, with particular attention to critical domains such as protocol registration (item 2), comprehensiveness of the literature search strategy (item 4), justification for excluded studies (item 7), risk of bias assessment of included studies (item 9), appropriateness of the meta-analytic methods (item 11), and consideration of publication bias (item 15). Based on the number and severity of critical flaws identified, each meta-analysis was rated as having high, moderate, low, or critically low confidence in its findings. Any disagreements between reviewers were resolved through discussion and consensus. AMSTAR-2 ratings were assigned according to the number of critical domains rated ‘No.’ Reviews with ≥1 critical flaw were downgraded to low or critically low confidence.

The risk of bias in the primary studies included within each meta-analysis was assessed using the tools employed by the original meta-analyses themselves. The most commonly used instruments were the SYRCLE risk of bias tool and the CAMARADES checklist. These tools evaluated key domains of bias, including selection bias, performance bias, detection bias, attrition bias, and reporting bias. The overall risk of bias for each meta-analysis—categorized as low, moderate, high, or unclear—was recorded as reported in the studies. If a meta-analysis utilized a custom or non-standard assessment tool, its specific criteria were documented accordingly.

To improve clarity, we distinguished the use of the SYRCLE and CAMARADES tools based on the model type and reporting structure of the original meta-analyses. Specifically, the SYRCLE tool was applied when the included meta-analysis assessed basic animal studies with heterogeneous outcomes such as behavioral scores, histological findings, or inflammatory markers. In contrast, the CAMARADES checklist was used when analyzing more structured preclinical models—particularly in neurological and cardiovascular studies—where endpoints such as infarct volume, mNSS, or neurobehavioral scores were commonly and consistently reported. In instances where both tools were used or a modified version was employed, we recorded that distinction accordingly in Table 4.

### 2.7 Data synthesis

Data were synthesized both narratively and quantitatively to comprehensively evaluate the therapeutic efficacy of MSC-EVs across various diseases, exosome sources, and outcome measures. The synthesis was structured to align with the objectives of the umbrella review, with a particular focus on therapeutic effectiveness, underlying mechanisms of action, and the methodological quality of the included meta-analyses.

A narrative synthesis was performed to describe the diversity of conditions addressed in the included studies, the types and tissue sources of MSC-EVs used, the animal models employed, and the administration routes applied. This synthesis also outlined the primary outcomes assessed, their consistency across studies, and the proposed mechanisms of action, such as anti-inflammatory, anti-apoptotic, and regenerative effects. Findings were organized into comprehensive tables and illustrative figures to facilitate interpretation and comparison. For instance, Table 3 presents a detailed summary of exosome-based therapies across different diseases and conditions, while visual aids such as bar graphs and merged heatmaps were used to depict data trends and outcome distributions.

In the quantitative synthesis, effect sizes, heterogeneity measures, and statistical significance were summarized based on the results reported in the included meta-analyses. Key metrics included SMD, WMD, HR, and OR, all accompanied by 95% confidence intervals. These metrics were typically reported for primary outcomes such as functional recovery scores, wound healing rates, or infarct volume reduction. Heterogeneity across studies was assessed using the I^2^ statistic, with values greater than 50% considered indicative of substantial variability. Where available, subgroup analyses or sensitivity analyses were reported to explore sources of heterogeneity. Publication bias was evaluated based on the original meta-analyses.

No additional meta-analyses were conducted within this umbrella review, as the aim was to synthesize and evaluate existing meta-analytic evidence rather than generate new pooled estimates. However, reported effect sizes were qualitatively summarized to identify therapeutic trends—for example, MSC-EVs demonstrated high efficacy in preclinical models of stroke and moderate effects in kidney transplantation models.

Because umbrella reviews synthesize findings from published meta-analyses without re-analyzing primary studies, we did not exclude individual studies on the basis of heterogeneity. Instead, we applied a rule-based classification: outcomes were labeled as High effectiveness only when SMD >1.5, p < 0.01, and I^2^ < 70% in ≥2 independent meta-analyses. Outcomes with I^2^ ≥ 70% were reclassified as Promising but heterogeneous and interpreted with caution. Sensitivity summaries were added to indicate whether conclusions remained robust after considering only meta-analyses with I^2^ < 70% and without AMSTAR-2 critical flaws.

Because this is an umbrella review, we did not exclude meta-analyses solely on the basis of high heterogeneity. Instead, we applied a rule-based classification: outcomes were labeled as High effectiveness only when SMD >1.5, p < 0.01, and I^2^ < 70% in ≥2 independent reviews. Outcomes with I^2^ ≥ 70% were reclassified as Promising but heterogeneous and interpreted with caution.

### 2.8 Subgroup and sensitivity analyses

Subgroup analyses reported within the included meta-analyses were extracted to identify factors that may influence the therapeutic efficacy of MSC-EVs. These analyses explored variations based on the source of exosomes—such as bone marrow-derived MSCs (BM-MSCs), adipose-derived MSCs (AD-MSCs), and human umbilical cord-derived MSCs (hUC-MSCs)—as well as animal model characteristics, including species and specific strains used in the experiments. Differences in disease models were also considered, such as contusion versus compression injury models for SCI, to evaluate how pathophysiological variations affect outcomes.

Additional subgroup variables included the route of MSC-EV administration and the timing and dosage of EV delivery. These factors were examined to determine their potential role in modulating therapeutic effectiveness across studies.

Sensitivity analyses conducted within the original meta-analyses were also summarized. These included procedures such as excluding studies with a high risk of bias to test the stability of the main findings, as well as statistical methods like trim-and-fill adjustments to evaluate the impact of publication bias. Together, these subgroup and sensitivity analyses provided important insights into the robustness and generalizability of MSC-EV therapy outcomes across different experimental conditions.

### 2.9 Ethical considerations

As this study involved no primary data collection or animal experimentation, ethical approval was not required. However, the review considered the ethical conduct of included studies, noting compliance with animal welfare regulations as reported by the meta-analyses.

### 2.10 Statistical software and tools

Several tools were employed to facilitate data management and ensure methodological consistency throughout the review process. EndNote 20 was used for reference management and to identify and remove duplicate records prior to screening. For data extraction and the creation of summary tables, Microsoft Excel was utilized, offering a structured format to capture and organize information efficiently. Additionally, RStudio was employed to generate heatmap graphs, enabling visual representation of data patterns and relationships derived from the synthesized findings.

No new statistical analyses were performed in this umbrella review, as its primary goal was to synthesize and interpret results from existing meta-analyses. However, statistical metrics reported in the included studies were carefully reviewed and verified for accuracy to ensure the reliability of the synthesized findings.

## 3 Results

This umbrella review synthesizes findings from 47 meta-analyses evaluating the therapeutic efficacy of MSC-EVs in preclinical animal models across a wide range of diseases and conditions (Table 2). The systematic search identified studies published between 2016 and 2025, covering diverse therapeutic applications, exosome sources, animal models, and outcome measures. The results are organized into subsections to provide a detailed overview of MSC-EV efficacy, mechanisms, sources, and methodological considerations.

**TABLE 2:** Descriptive summary of meta-analyses evaluating mesenchymal stem cell-derived extracellular vesicles in preclinical studies.

Authors, reference	Year	Journal	Number of studies	Disease/ Condition	Intervention	Exosome source	Animal model	Outcomes	Main findings	Risk of bias assessment
Aghayan et al. (2024)	2024	Stem Cell Rev Rep	30	Sepsis	MSC-EVs	BM, UC, AT, placenta	Mice, rats, sheep	Survival, organ function, cytokines	Improved survival (HR 0.33, 95% CI 0.27–0.41), reduced organ damage, modulated inflammation. BM-EVs most effective	High certainty (GRADE); low risk (SYRCLE, novel tool)
Bailey et al. (2022)	2022	Stem Cell Rev Rep	10	Diabetic wounds	MSC-EVs	BM, UC, AT, synovia, others	Mice, rats	Wound closure, angiogenesis, inflammation	Enhanced closure (SMD 5.48, 95% CI 3.55–8.13), angiogenesis, reduced inflammation. RNA-enriched EVs more effective	Unclear risk (SYRCLE); unclear blinding, randomization
Bernardi et al. (2025)	2025	Mol Neurobiol	35	Ischemic stroke	MSC-EVs	BM, UC, AT	Rodents, monkeys, ewes	Microglia, cytokines	Reduced inflammation, Iba1^+^, TNF-α	Moderate-high risk (SYRCLE); 67% moderate, 33% high
Chen et al. (2024)	2024	Heliyon	55	Traumatic brain injury	MSC-EVs	BM	Mice, rats	Neurological scores, lesion volume	Improved mNSS, MWM; reduced lesion volume	Moderate risk (CAMARADES, SYRCLE); mean score 5.75
Chen et al. (2023)	2023	Heliyon	7	Intrauterine adhesion	Stem cells EVs	UC, BM, AT, others	Rats, rabbits	Fibrosis, endometrial repair	Reduced fibrosis, increased embryo number	High risk (SYRCLE); publication bias (Egger’s p = 0.005)
Dai et al. (2025)	2025	Lipids Health Dis	14	NAFLD, NASH	MSC-EVs	UC, AT, BM	Mice, rats	Liver markers, cytokines	Reduced AST, ALT, TG, NAS, oxidative stress	High risk (SYRCLE); poor methodology
Fang et al. (2023)	2023	J Pers Med	39	Liver diseases	MSC-EVs	BM, UC, AT, ESCs	Mice, rats	Liver function, histology, cytokines	Reduced damage, inflammation	Moderate risk (CAMARADES); high heterogeneity
Fang et al. (2022)	2022	Transplant Rev	7	Kidney transplantation	MSC-EVs, immune cell-EVs	BM, AT	Mice, rats	Graft survival, SCr, BUN	Immune-EVs more effective than MSC-EVs	Moderate-high risk (SYRCLE)
Firouzabadi et al. (2024a)	2024	Stem Cell Rev Rep	19	Asthma	MSC-EVs	BM, UC, AT, iPSC	Mice, rats	Inflammation, airway responsiveness	Reduced inflammation, hyper-responsiveness. Dose/timing critical	Unclear risk (SYRCLE); publication bias (Egger’s p < 0.05)
Firouzabadi et al. (2024b)	2024	J Ovarian Res	29	Primary ovarian insufficiency	MSC-EVs	UC, BM, AT, others	Mice, rats	Follicle count, hormones, pregnancy	Improved follicle number, hormones, pregnancy rate	Low quality (SYRCLE); high heterogeneity, bias
Gunjan et al. (2024)	2024	Mol Cell Probes	8	Diabetic wound healing	MSC-EVs	BM, UC, AT	Mice, rats	Wound closure, angiogenesis	Enhanced closure, angiogenesis	High risk (SYRCLE); high heterogeneity, bias
He et al. (2022)	2022	Stem Cell Res Ther	9	Subarachnoid hemorrhage	MSC-EVs, MSCs	BM, UC	Mice, rats	Neurobehavior, brain edema	EVs outperformed MSCs in neuroprotection	High risk (CAMARADES); high heterogeneity
He et al. (2023)	2023	Stem Cell Res Ther	11	Osteoporosis	MSC-EVs, ESC-EVs	UC, BM, AT, ESCs	Mice, rats	Bone mass, structure	Increased BMD, microarchitecture	High risk (SYRCLE); low randomization, blinding
Hickson et al. (2021)	2021	Stem Cells Transl Med	40	Diabetic kidney disease	MSC-EVs, MSCs	BM, UC, AT	Mice, rats, shrews	Renal function, inflammation	Reduced creatinine, fibrosis, inflammation	Low risk (SYRCLE); strong reporting
Himanshu et al. (2025)	2025	Biochem Biophys Rep	15	Chronic kidney disease	MSC-EVs	BM, UC, AT	Mice, rats	SCr, BUN, renal damage	Reduced SCr, BUN, inflammation	Moderate risk (SYRCLE); moderate heterogeneity
Jabermoradi et al. (2025)	2024	Arch Acad Emerg Med	65	SCI	MSC-EVs	BM, UC, AT	Mice, rats	Locomotion, neural markers	Improved motor recovery, reduced apoptosis	Moderate risk (SYRCLE); low bias for most outcomes
Kirkham et al. (2022)	2022	Stem Cell Rev Rep	13	Bone injury	MSC-EVs	BM, UC, AT, dental	Mice, rats	BV/TV, bone formation	Improved BV/TV, bone formation. Modified EVs no added benefit	Unclear risk (SYRCLE); no publication bias
Liu et al. (2020a)	2020	Stem Cell Res Ther	31	Acute kidney injury	MSC-EVs	BM, UC, AT, others	Mice, rats	SCr, BUN, inflammation	Improved SCr, BUN, reduced inflammation	Unclear risk (CAMARADES); publication bias adjusted
Liu et al. (2024)	2024	Front Cell Dev Biol	25	Osteosarcoma	MSC-EVs	BM, unspecified	Mice	Tumor volume, weight	Engineered EVs more effective; macrophage EVs promoted growth	Unclear risk (SYRCLE); possible bias
Lou et al. (2025)	2025	Stem Cell Res Ther	20	Erectile dysfunction	MSC-EVs	MSC, AT, UC	Rats	ICP/MAP, nNOS, eNOS	Improved function, no source/model difference	Moderate-high quality (SYRCLE); publication bias
Lv et al. (2025)	2025	Front Pharmacol	14	Kidney fibrosis	MSC-EVs	UC	Rats, mice	SCr, BUN, fibrosis	Reduced fibrosis, inflammation; increased E-Cadherin	Low-moderate risk (SYRCLE); no bias
Mou et al. (2025)	2025	Front Neurol	21	Acute SCI	MSC-EVs	BM, UC, AT, placenta	Rats	BBB scores	Improved locomotor recovery, reduced inflammation	Low risk (SYRCLE); slight publication bias
Nowak et al. (2022)	2022	Stem Cell Rev Rep	35	Chronic kidney disease	MSC-EVs	BM, UC, AT	Mice, rats	SCr, GFR, fibrosis	Improved renal outcomes, miRNA-mediated	Unclear risk (CAMARADES); bias adjusted
Shang et al. (2024)	2024	Cytotherapy	40	Traumatic SCI	Stem cell-EVs	BM, AT, UC, NSCs	Rats	BBB scores	Improved motor function (WMD 1.58–4.54); NSCs-EVs most effective	Unclear risk (SYRCLE); publication bias
Soltani et al. (2024)	2024	Stem Cell Rev Rep	20	Diabetic wounds	ADSC-EVs	AT	Mice, rats	Wound closure, angiogenesis	Enhanced closure (SMD 4.22, 95% CI 3.07–5.36), angiogenesis	Unclear risk (SYRCLE); unclear bias details
Tieu et al. (2021)	2021	J Extracell Vesicles	11	Respiratory diseases	MSC-EVs	BM, UC, AT	Preclinical models	Inflammation, fibrosis	Improved acute/chronic respiratory outcomes	Not specified
Wang et al. (2024)	2024	Stem Cells Int	12	Hemorrhagic stroke	Stem cell-EVs	MSC, AT	Mice, rats	Neurobehavioral scores	Improved SAH, chronic ICH outcomes (SMD -3.49, 2.38)	High quality (CAMARADES); publication bias
Wang et al. (2020)	2020	Respir Res	17	Acute lung injury/ARDS	MSC-EVs	BM, UC, AT, neural	Mice, rats, pigs	Lung injury, survival	Reduced injury (SMD -4.02), improved survival (OR 6.45)	Moderate-high heterogeneity; bias not detailed
Wang et al. (2025)	2025	Front Pharmacol	28	Knee osteoarthritis	MSC-EVs	BM, UC, AT, others	Rats	Cartilage repair, inflammation	Improved repair (OARSI SMD -2.97), reduced inflammation	Moderate quality (SYRCLE); unclear bias reporting
Wendt et al. (2018)	2018	Sci Rep	43	Cardiovascular diseases	EVs	MSC, cardiac cells	Rodents, pigs	Cardiac function, inflammation	Reduced injury, improved function, angiogenesis	Low reporting bias; unclear EV characterization
Xu et al. (2024)	2024	Front Pharmacol	38	Ischemic stroke	Stem cell-EVs	BM, UC, AT, others	Mice, rats	Infarct volume, mNSS	BMSC-EVs most effective; engineered EVs enhanced efficacy	Moderate risk (SYRCLE); publication bias
Xun et al. (2022)	2022	Front Immunol	12	Multiple sclerosis	MSC-EVs	BM, UC, AT, dental	Mice, rats	Clinical score	Improved symptoms (SMD -2.17); PDLSCs most effective	Unclear risk (SYRCLE); insufficient reporting
Yang et al. (2023a)	2023	Neural Regen Res	49	SCI, TBI	MSC-EVs	BM, UC, AT, placenta	Mice, rats	BBB, mNSS, Foot Fault	Improved SCI (SMD 4.46), TBI outcomes; reduced inflammation	Moderate risk (SYRCLE); publication bias
Yang et al. (2022)	2022	Front Neurosci	13	Spinal cord injury	MSC-EVs, miRNA-EVs	BM, UC, AT	Rats	BBB scores	miRNA-EVs improved motor function; contusion models better	Low risk (SYRCLE); publication bias
Yang et al. (2023b)	2023	Front Neurosci	20	Traumatic brain injury	EVs	MSC, astrocytes, NSCs	Mice, rats	mNSS, MWM	Astrocyte-EVs most effective; improved mNSS, MWM.	Uneven quality (SYRCLE); no bias for mNSS.
Ye et al. (2024)	2024	Front Mol Neurosci	30	SCI	BMSC-EVs	BM	Rats	BBB, inflammation, apoptosis	Improved outcomes; dose-response correlation	High risk (SYRCLE); unclear randomization, blinding
Yi and Wang (2021)	2021	Open Med	35	Acute SCI	EVs	MSC, HUVECs, PC12	Mice, rats	BBB, BMS	Improved locomotor recovery; intrathecal better	Low risk (SYRCLE); publication bias in BBB.
Yue et al. (2024)	2024	Front Endocrinol	21	Type II diabetic wounds	MSC-EVs	AT, BM, UC, others	Mice, rats	Wound closure, inflammation	Improved healing (SMD >3), reduced inflammation	Unclear risk (SYRCLE); publication bias
Zhang et al. (2016a)	2016	Exp Ther Med	13	Acute kidney injury	MSC-EVs	BM, UC, AT	Rodents	SCr	EVs more effective than CM; early administration better	High heterogeneity; no publication bias
Zhang et al. (2016b)	2016	Stem Cells Int	6	Myocardial I/R injury	MSC-EVs	ESC-MSC, others	Mice, pigs	Cardiac function	Improved EF, FS; EVs better than CM.	High heterogeneity; no bias analysis
Zhang et al. (2022)	2022	Neural Plast	24	Cerebral I/R injury	MSC-EVs	BM, UC, AT	Mice, rats	Infarct volume, neuro score	Reduced infarct, improved neurology	Moderate quality (SYRCLE); publication bias
Zhang et al. (2025)	2025	Brain Res Bull	73	Ischemic stroke	MSC-EVs	BM, UC, AT	Mice, rats	Infarct volume, mNSS	Reduced infarct, improved function (P < 0.01)	High quality (CAMARADES); median score 8/10
Zhou et al. (2023a)	2023	BMC Oral Health	11	Periodontitis	MSC-EVs	BM, dental	Mice, rats	BV/TV, CEJ-ABC	Improved BV/TV, reduced CEJ-ABC.	Unclear risk (SYRCLE); no bias in key metrics
Zhou et al. (2025)	2025	J Orthop Surg Res	17	Periodontitis	MSC-EVs	Dental, BM, UC	Mice, rats, beagles	BV/TV, BMD, CEJ-ABC	Improved BV/TV (SMD 13.99), BMD; no Tb.Th effect	Unclear risk (SYRCLE); high heterogeneity
Zhou et al. (2024)	2024	Front Pharmacol	18	Liver fibrosis	MSC-EVs	BM, UC, AT	Mice, rats	Liver function, fibrosis	Improved function, fibrosis; EVs + drugs better	Mixed risk (Cochrane); high heterogeneity
Zhou et al. (2023b)	2023	Stem Cell Rev Rep	28	POI, IUA	MSC-EVs	BM, UC, menstrual	Mice, rats	AMH, endometrial thickness	Improved AMH, endometrium; better with scaffolds	Unclear risk (SYRCLE); bias for AMH in POI.
Zhu et al. (2025)	2025	J Transl Med	83	Skin regeneration	MSC-EVs	BM, UC, AT, others	Mice, rats	Wound closure, collagen	Improved closure, collagen; ApoSEVs best	Low quality (MISEV2023); high heterogeneity

Abbreviations: MSC-EVs, Mesenchymal stem cell-derived extracellular vesicles; BM, bone marrow; UC, umbilical cord; AT, adipose tissue; SCr, Serum creatinine; BUN, blood urea nitrogen; BBB, basso, Beattie, Bresnahan; mNSS, modified neurological severity score; MWM, morris water maze; SMD, standardized mean difference; HR, hazard ratio; CI, confidence interval; BV/TV, Bone volume/total volume; CEJ-ABC, Cementoenamel junction-alveolar bone crest; NAFLD, Non-alcoholic fatty liver disease; NASH, Non-alcoholic steatohepatitis; IUA, intrauterine adhesion; POI, primary ovarian insufficiency; TBI, traumatic brain injury; SCI, spinal cord injury.

### 3.1 Therapeutic efficacy across diseases

**FIGURE 2:**
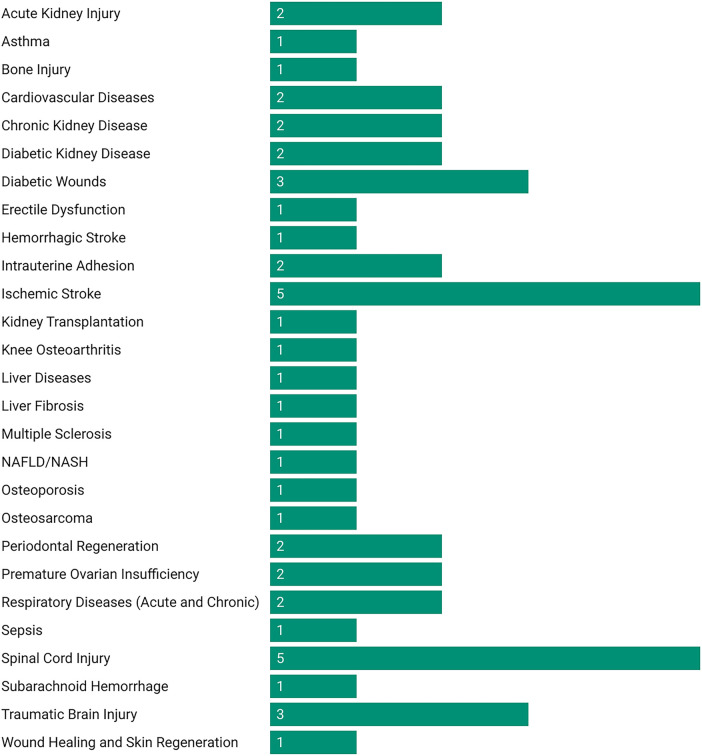
Number of meta-analyses evaluating MSC-EV therapies in preclinical models by disease category.

**FIGURE 3:**
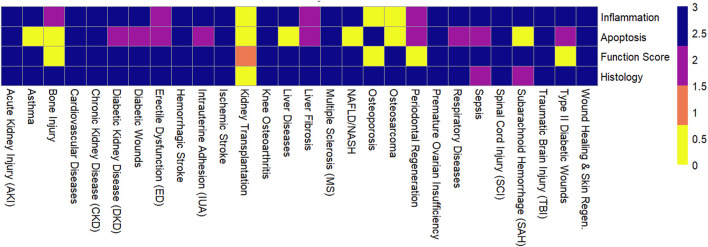
Effectiveness of mesenchymal stem cell-derived extracellular vesicles across outcomes for various diseases.

MSC-EVs demonstrated high therapeutic efficacy across most evaluated diseases, with consistent improvements in functional, histological, and molecular outcomes (Figure 2). The following summarizes key findings by disease category (Figure 3; Supplementary Table S1). MSC-EVs consistently reduced inflammation and apoptosis, while enhancing functional scores and histological repair. Effectiveness was high across most conditions, with bone marrow-derived MSC-EVs (BMSC-EVs) and preconditioned EVs showing superior results, though heterogeneity was moderate to high and risk of bias varied. The classification of therapeutic effectiveness into “high” and “moderate” was based on reported meta-analytic metrics. “High” effectiveness was assigned to outcomes with standardized mean difference (SMD) > 1.5, p < 0.01, and low-to-moderate heterogeneity (I^2^ < 70%) observed in at least two independent meta-analyses. “Moderate” effectiveness was applied to outcomes with SMD values between 0.8 and 1.5 or when heterogeneity exceeded 70%.

MSC-EVs exert their therapeutic effects through a range of interconnected biological mechanisms. These mechanisms contribute to the regenerative and protective roles of MSC-EVs in various pathological conditions.

One of the most prominent mechanisms is the anti-inflammatory effect. MSC-EVs were consistently shown to downregulate proinflammatory cytokines such as tumor necrosis factor-alpha (TNF-α), interleukin-1 beta (IL-1β), and interleukin-6 (IL-6), while simultaneously upregulating anti-inflammatory mediators including interleukin-10 (IL-10) and transforming growth factor-beta 1 (TGF-β1). These immunomodulatory effects were observed across multiple disease models, particularly in stroke, SCI (SCI), acute kidney injury (AKI), and asthma.

Anti-apoptotic effects were also widely reported. MSC-EVs reduced markers of apoptosis, such as caspase-3 and Bax, in neurological, renal, and cardiovascular models. By inhibiting apoptotic pathways, MSC-EVs helped preserve tissue integrity and cell viability in damaged organs.

Functional improvements were another key therapeutic outcome, with enhanced performance in disease-specific scoring systems such as the Basso, Beattie, Bresnahan (BBB) score for SCI, the modified Neurological Severity Score (mNSS) for stroke, and the Osteoarthritis Research Society International (OARSI) score for joint degeneration. These improvements were largely attributed to mechanisms such as neuroregeneration, angiogenesis, and overall tissue repair facilitated by MSC-EVs.

Finally, histological improvements supported the regenerative potential of MSC-EVs. Across studies, MSC-EVs were shown to stimulate collagen deposition, promote angiogenesis and neurogenesis, and reduce fibrosis, lesion size, and tissue damage. These histological changes were particularly evident in models of wound healing, liver fibrosis, and kidney disease, underscoring the broad-spectrum therapeutic action of MSC-EVs across organ systems.

Across conditions such as ischemic stroke, diabetic wounds, SCI, and acute kidney injury, MSC-EVs significantly reduced inflammation, apoptosis, and tissue damage while enhancing functional recovery and histological repair (Table 3). BMSC-EVs, adipose-derived MSC-EVs (ADSC-EVs), and preconditioned EVs showed superior efficacy in conditions like ischemic stroke, diabetic wounds, and multiple sclerosis, with notable improvements in neurovascular repair, wound closure, and clinical scores. However, effectiveness was low in kidney transplantation, where MSC-EVs showed no significant benefit. Consistency across studies was moderate (I^2^ = 23–95%) for most conditions, with high heterogeneity in bone injury (I^2^ = 97–98%) and acute kidney injury (I^2^ = 96%), likely due to variability in animal models, exosome sources, and administration methods. For disease areas where heterogeneity was very high (I^2^ ≥ 70%), such as bone injury and acute kidney injury, the results were reclassified as Promising but heterogeneous. While these conditions showed large effect sizes, the variability across studies limits certainty in the pooled estimates. For such disease areas with I^2^ ≥ 70%, outcomes were downgraded to Promising but heterogeneous. While effect sizes were large, the variability across studies limits the certainty of pooled estimates.

**TABLE 3:** Comprehensive summary of mesenchymal stem cell-derived extracellular vesicles-based therapies across diseases and conditions.

Disease/Condition	Number of reviews	Animal models	Exosome source^a^	Main outcomes	Effectiveness^b^	Consistency (I^2^)
Acute Kidney Injury	1	Mice, rats	BM-MSCs, UC-MSCs, AD-MSCs, others	Reduced SCr (MD 0.93, 95% CI 0.67–1.20), BUN, TNF-α; increased IL-10; improved renal function	Promising but heterogeneous (EVs > CM)	Low (96%)
Asthma	1	Mice, rats	BM-MSCs, UC-MSCs, AD-MSCs, iPSC-MSCs	Reduced IL-4, eosinophils, collagen, AHR; increased IL-10	Promising but heterogeneous	Moderate (72%–93%)
Bone Injury	1	Mice, rats	BM-MSCs, UC-MSCs, AD-MSCs, dental MSCs	Increased BV/TV (22.2%), NBF (26.1%), mTOR/AKT, BMP2 activation	Promising but heterogeneous	Low (97%–98%)
Cardiovascular Diseases	2	Mice, rats, pigs	BM-MSCs, UC-MSCs, AD-MSCs, CPCs, ESCs	Reduced infarct size (SMD -5.87, 95% CI -7.07 to −4.67), apoptosis; improved EF (SMD 1.57, 95% CI 0.86–1.26), angiogenesis	Promising but heterogeneous	Moderate (86%–94%)
Chronic Kidney Disease	2	Mice, rats	BM-MSCs, UC-MSCs, AD-MSCs	Reduced fibrosis, inflammation; improved GFR, renal function	Promising but heterogeneous	Moderate (67%–95%)
Diabetic Kidney Disease	2	Mice, rats, shrews	BM-MSCs, UC-MSCs, AD-MSCs, others	Reduced SCr, BUN, fibrosis; increased IL-10; improved histology	Promising but heterogeneous	Moderate (60%–94%)
Diabetic Wounds	2	Mice, rats	AD-MSCs, BM-MSCs, UC-MSCs, others	Enhanced closure (SMD 4.22, 95% CI 3.07–5.36), angiogenesis (SMD 9.27, 95% CI 4.70–13.83), collagen	Promising but heterogeneous (ADSC-EVs, ApoSEVs best)	Moderate-High (39%–88%)
Erectile Dysfunction	1	Rats	MSCs, AD-MSCs, UC-MSCs	Improved ICP/MAP, NOS, smooth muscle ratio	Promising but heterogeneous	Moderate (74%–86%)
Hemorrhagic Stroke	1	Mice, rats	BM-MSCs, AD-MSCs, UC-MSCs	Improved neurobehavior in SAH (SMD -3.49, 95% CI -4.23 to −2.75), chronic ICH; reduced apoptosis, inflammation	Promising but heterogeneous (SAH, chronic ICH)	Moderate (23%–92%)
Intrauterine Adhesion	1	Rats, rabbits	UC-MSCs, BM-MSCs, AD-MSCs, others	Increased endometrial thickness (WMD 132.36, 95% CI 118.99–145.74), glands; reduced fibrosis	Promising but heterogeneous (HA/collagen enhanced)	Moderate (54%–95%)
Ischemic Stroke	4	Mice, rats, monkeys, ewes	BM-MSCs, UC-MSCs, AD-MSCs, NSCs, others	Reduced infarct volume (SMD -3.76, 95% CI -4.22 to −3.29), mNSS; enhanced neurovascular repair	Promising but heterogeneous (BMSC-EVs best)	Moderate (43%–92%)
Kidney Transplantation	1	Mice, rats	BM-MSCs, AD-MSCs	Prolonged graft survival; MSC-EVs not significant	Low (MSC-EVs)	Low (91%–94%)
Knee Osteoarthritis	1	Rats	BM-MSCs, UC-MSCs, AD-MSCs, others	Improved OARSI score (SMD -2.97, 95% CI -3.62 to −2.31), collagen II; reduced IL-1β, TNF-α	Promising but heterogeneous (UMSC-EVs best)	Moderate (0%–81%)
Liver Diseases	1	Mice, rats	BM-MSCs, UC-MSCs, AD-MSCs, others	Improved liver enzymes, reduced fibrosis, inflammation	Promising but heterogeneous	Moderate-High (0%–80%)
Liver Fibrosis	1	Mice, rats	BM-MSCs, UC-MSCs, AD-MSCs	Reduced collagen (SMD -2.92, 95% CI -4.76 to −1.08), α-SMA; improved ALT, AST	Promising but heterogeneous (ADSC-EVs, EV + drugs best)	Moderate (70%–91%)
Multiple Sclerosis	1	Mice, rats	BM-MSCs, UC-MSCs, AD-MSCs, PDLSCs	Improved clinical score (SMD -2.17, 95% CI -3.99 to −0.34); reduced inflammation	Promising but heterogeneous (PDLSCs best)	Moderate (84%)
NAFLD/NASH	1	Mice, rats	UC-MSCs, AD-MSCs, BM-MSCs	Reduced liver fat, inflammation; increased SOD	Promising but heterogeneous	Not reported
Osteoporosis	1	Mice, rats	UC-MSCs, BM-MSCs, AD-MSCs	Improved BMD, bone microstructure	Promising but heterogeneous	Low-Moderate (71%–87%)
Osteosarcoma	1	Mice	BM-MSCs, AD-MSCs, macrophages	Reduced tumor volume; macrophage-EVs most effective	Promising but heterogeneous	Moderate (40%–70%)
Periodontal Regeneration	2	Mice, rats, beagles	BM-MSCs, UC-MSCs, dental MSCs	Increased BV/TV (WMD 14.07, 95% CI 6.73–21.41), BMD; reduced CEJ-ABC	Promising but heterogeneous (preconditioned EVs best)	Moderate (36%–99%)
Premature Ovarian Insufficiency	1	Mice	BM-MSCs, UC-MSCs, AD-MSCs, others	Improved AMH (SMD 5.39, 95% CI 3.43–7.36), E2; reduced FSH	Promising but heterogeneous	Moderate (76%–95%)
Respiratory Diseases	2	Mice, rats, pigs	BM-MSCs, UC-MSCs, AD-MSCs	Reduced lung injury (SMD -4.02, 95% CI -5.28 to −2.23); improved survival (OR 6.45, 95% CI 2.78–14.97)	Promising but heterogeneous	Moderate (67%–95%)
Sepsis	1	Mice, rats, sheep	BM-MSCs, UC-MSCs, AD-MSCs	Improved survival, organ function; reduced TNF-α, IL-6	Promising but heterogeneous	Moderate (Not reported)
Spinal Cord Injury	4	Mice, rats	BM-MSCs, UC-MSCs, AD-MSCs, NSCs	Improved BBB score (WMD 3.47, 95% CI 3.31–3.63); reduced inflammation, apoptosis	Promising but heterogeneous (BMSC-EVs, NSC-EVs best)	Moderate (75%–81%)
Subarachnoid Hemorrhage	1	Mice, rats	BM-MSCs, UC-MSCs	Improved neurobehavior; reduced brain edema	Promising but heterogeneous	Moderate (58%–89%)
Traumatic Brain Injury	2	Mice, rats	BM-MSCs, UC-MSCs, AD-MSCs, astrocytes	Improved mNSS (SMD -4.48), MWM; reduced inflammation, lesion volume	Promising but heterogeneous (AEVs best early)	Moderate (76%–94%)
Wound Healing/Skin Regeneration	1	Mice, rats	BM-MSCs, UC-MSCs, AD-MSCs, others	Improved closure (SMD 3.60, 95% CI 3.23–3.96), angiogenesis, collagen	Promising but heterogeneous (ApoSEVs, ADSC-EVs best)	Moderate (82%–85%)

Abbreviations: MSC-EVs, Mesenchymal stem cell-derived extracellular vesicles; BM-MSCs, Bone marrow MSCs; UC-MSCs, Umbilical cord MSCs; AD-MSCs, Adipose tissue MSCs; SCr, Serum creatinine; BUN, blood urea nitrogen; BBB, basso, Beattie, Bresnahan; mNSS, modified neurological severity score; MWM, morris water maze; SMD, standardized mean difference; WMD, weighted mean difference; CI, confidence interval; BV/TV, Bone volume/total volume; CEJ-ABC, Cementoenamel junction-alveolar bone crest; AHR, Airway hyper-responsiveness; AMH, Anti-Müllerian hormone; NAFLD, Non-alcoholic fatty liver disease; NASH, Non-alcoholic steatohepatitis; ICH, intracerebral hemorrhage; SAH, subarachnoid hemorrhage.

^a^ Administration routes are summarized by disease model; CNS, models frequently employed intrathecal or intranasal delivery, whereas local injection/hydrogel strategies were common in wound and periodontal models.

^b^ High effectiveness required SMD >1.5, p < 0.01, and I2 < 70% in ≥2 independent meta-analyses. Outcomes with I2 ≥ 70% were reclassified as Promising but heterogeneous.

Administration routes varied substantially across conditions. Intravenous delivery was the predominant method in most disease models, including renal and hepatic injury. For CNS models such as spinal cord injury and ischemic stroke, intrathecal, intranasal, or intracerebroventricular administration was frequently used and, in some cases, demonstrated greater efficacy by enabling direct delivery across the blood–brain barrier. For local diseases such as diabetic wounds and periodontal regeneration, local injections or hydrogel/scaffold-based delivery systems were commonly applied, supporting tissue retention and enhancing therapeutic benefit. These findings, summarized in Table 4, indicate that administration route is an important factor influencing MSC-EV efficacy and should be tailored to the target organ and disease.

**TABLE 4:** Overview of mesenchymal stem cell-derived extracellular vesicle (MSC-EV) dosing strategies, sources, administration routes, dose units, and evaluation of dose-response effects in preclinical meta-analyses.

Author(s) (Year) (references)	MSC source	EV dose	Administration route	Dose unit	Dose-response studied
Aghayan et al. (2024)	BM-MSCs UC-MSCs AD-MSCs	2 μg–300 µg 1 × 10^8^ to 1 × 10^11^ particles	IV IP IT	µg Particle number	Not
Bailey et al. (2022)	Various tissues	10–200 µg 1.83 × 10^10^–5.22 × 10^10^ particles	Hydrogel Intradermal SC Direct injection	µg Particle number	Not
Bernardi et al. (2025)	BM-MSCs UC-MSCs AD-MSCs	Single or multiple bolus various time points (0–168 h post-ischemia)	IV IC IN Intraarterial Others	Not uniformly reported (µg or particle number)	Yes
Chen et al. (2023)	BM-MSCs	20–100 μg EV protein (injected daily for 3–7 days) 1.6–4.2 × 10^8^ particles	IV IN Local injection	µg Particle number	Partially
Chen et al. (2024)	BM-MSCs UC-MSCs AD-MSCs uMSCs MenSCs	25–100 µg (mass) 0.25–0.5 mL (volume) 2.13 × 10^7^· particles	Intrauterine IV	µg mL Particle number	Not
Dai et al. (2025)	BM-MSCs UC-MSCs AD-MSCs	30–200 µg or ∼1 × 10^9^ particles	IV	µg Particle number	Yes
Fang et al. (2022)	BM-MSCs AD-MSCs	9.6–11.7 µg 1–1.4 × 10^9^ particles 10 µg	IV Intrasplenic	µg Particle number	Not
Fang et al. (2023)	BM-MSCs UC-MSCs AD-MSCs ESC-MSCs	100–500 µg per injection	IV Local injection	µg	Not
Firouzabadi et al. (2024a)	BM-MSCs UC-MSCs AD-MSCs iPSC-MSCs	20–100 µg 1 × 10^9^ to 5 × 10^5^ particles mostly single or 2-dose regimens	IV IN	µg Particle number	Not
Firouzabadi et al. (2024b)	BM-MSCs UC-MSCs AD-MSCs iPSC-MSCs AF-MSC	10 μg–400 µg total dose ranged from 10 to 1200 µg	IV Intra-ovarian IP	µg Particle number	Not
Gunjan et al. (2024)	BM-MSCs UC-MSCs AD-MSCs	Varied from 30 to 150 µg ∼1.8 × 10^10^ to 5.2 × 10^10^ particles	Local SC Hydrogel	µg Particle number	Not
He et al. (2022)	BM-MSCs UC-MSCs	100 µg 200–400 µg 2 × 10^5^ MSCs	IV ICV IN	µg Particle number	Not
He et al. (2023)	BM-MSCs UC-MSCs AD-MSCs ESC-MSCs	20–200 µg ∼1–5 × 10^9^	IV Local injection	µg Particle number	Yes
Hickson et al. (2021)	BM-MSCs UC-MSCs AD-MSCs	40–200 µg protein per dose 1 × 10^9^–1 × 10^11^ particles	IV IP	µg Particle number	Not
Himanshu et al. (2025)	BM-MSCs UC-MSCs AD-MSCs PSC-MSCs	20–250 µg protein 1 × 10^5^–1 × 10^11^ particles	IV IM IP IC	µg Particle number	Not
Jabermoradi et al. (2025)	BM-MSCs UC-MSCs AD-MSCs	20–150 µg 5 × 10^9^ × 10^10^ particles	IV IT Direct spinal cord	µg Particle number	Yes
Kirkham et al. (2022)	BM-MSCs AD-MSCs UC-MSCs Dental MSCs	1–200 µg or 1–1000 × 10^8^ particles	Local implantation (hydrogel/scaffold) Local injection IV	µg Particle number	Not
Liu et al. (2020a)	BM-MSCs UC-MSCs WJ-MSCs AD-MSCs UVECs	100 µg (20–200 µg) 2–5 × 10^10^ particles	IV Renal capsule	µg Particle number	Yes
Liu et al. (2024)	BM-MSCs UC-MSCs AD-MSCs	30–150 µg 1 × 10^9^ × 10^11^ particles per dose	IV IN ICV	µg Particle number	Not
Lou et al. (2025)	BM-MSCs UC-MSCs AD-MSCs	25–100 µg per dose occasional studies used 1 × 10^10^ particles	Corpus cavernosum IV	µg Particle number	Not
Lv et al. (2025)	UCB-MSCs	1 × 10^4^ to 1 × 10^6^	IV IP	Particle number	Yes
Mou et al. (2025)	BM-MSCs UC-MSCs AD-MSCs Placenta- MSCs	20–200 µg 1 × 10^9^ to 2 × 10^10^ particles per dose	IV IT Local injection	µg Particle number	Not
Nowak et al. (2022)	BM-MSCs UC-MSCs AD-MSCs	30–200 µg 1 × 10^9^–2 × 10^10^ particles per dose	IV Renal capsule	µg Particle number	Not
Shang et al. (2024)	BM-MSCs AD-MSCs UC-MSCs NSCs	40–200 µg per dose	IV	µg	Not
Soltani et al. (2024)	AD-MSCs	10–200 µg per dose 1 × 10^9^–2 × 10^10^ particles; mostly single dose	SC Hydrogel/dressing delivery	µg Particle number	Yes
Tieu et al. (2021)	BM-MSCs AD-MSCs UC-MSCs	50–250 µg 1 × 10^9^ × 10^11^ particles	SC Topical IV	µg Particle number	Partially
Wang et al. (2020)	BM-MSCs UC-MSCs AD-MSCs WJ-MSCs	10–100 µg protein 1 × 10^5^–10^8^ particles	IV IT Intratracheal	µg Particle number	Not
Wang et al. (2024)	BM-MSCs UC-MSCs AD-MSCs	20–400 µg protein 3 × 10^6^ cells equivalent	IV Intraventricular	µg Cell-equivalent	Yes
Wang et al. (2025)	UC-MSCs BM-MSCs	30–200 µg 1 × 10^9^–1 × 10^10^ particles per injection	IV IN	µg Particle number	Not
Wendt et al. (2018)	BM-MSCs	30–100 µg per injection	IV Local injection	µg	Not
Xu et al. (2024)	BM-MSCs UC-MSCs AD-MSCs iPSC-MSCs	10–300 µg 2 × 10^6^–3 × 10^11^ particles 10–200 μg/kg 800 ng-100 µg	IV IN Intracerebral	µg µg/kg Particle number	Yes
Xun et al. (2022)	UC-MSCs AD-MSCs	100–300 µg 1 × 10^9^ to 2 × 10^10^ particles	IV IN	µg Particle number	Partially
Yang et al. (2022)	BM-MSCs AD-MSCs	100–700 µg	IV Intrathecal	µg	Yes
Yang et al. (2023a)	BM-MSCs UC-MSCs AD-MSCs NSCs	3–200 µg 3 × 10^10^ particles 1.5 × 10^6^ cells	IV Intraventricular Retroorbital	µg Particle number Cell-based equivalent	Yes
Yang et al. (2023b)	BM-MSCs UC-MSCs AD-MSCs Placenta-MSCs	100 µg 20–400 µg 1 × 10^9^–3 × 10^11^ particles	IV IT IN Retroorbital ICV	µg Particle number	Yes
Ye et al. (2024)	BM-MSCs	100 µg per injection 100–500 µg	IV IT	µg	Yes
Yi and Wang (2021)	BM-MSCs UC-MSCs AD-MSCs NSCs EF-MSCs	10–700 µg per injection 200 μg/mL 5 × 10^10^ particles	IV IT IN Intracerebral Retroorbital	µg µg/mL Particle number	Yes
Yue et al. (2024)	BM-MSCs UC-MSCs AD-MSCs	10–200 µg per injection 1 × 10^9^ to 2 × 10^10^ particles	SC Intradermal Hydrogel-assisted topical delivery	µg Particle number	Not
Zhang et al. (2016a)	BM-MSCs UC-MSCs AD-MSCs	10–200 µg per dose	IV	µg	Not
Zhang et al. (2016b)	ESC-MSCs BM-MSCs	100–200 µg	IV	µg CM-equivalent	Not
Zhang et al. (2022)	BM-MSCs UC-MSCs AD-MSCs	50–200 µg 1 × 10^9^–2 × 10^10^ particles	IV IN	µg Particle number	Yes
Zhang et al. (2025)	BM-MSCs UC-MSCs AD-MSCs	100 µg Up to 300 µg	IV Local cerebral injection	µg	Yes
Zhou et al. (2023a)	BM-MSCs Dental MSCs	100–300 µg 1 × 10^9^–2 × 10^10^ particles	Local gingival injection IV Scaffold implantation	µg Particle number	Not
Zhou et al. (2023b)	BM-MSCs Dental MSCs	100 µg 1.5 × 10^9^ particles	Local injection	µg Particle number	Not
Zhou et al. (2024)	BM-MSCs UC-MSCs AD-MSCs TMSC AMSCs	40–400 µg 100–250 µg	IV IP Liver lobe injection	µg	Yes
Zhou et al. (2025)	BM-MSCs UC-MSCs MenSCs	10–100 µg per injection	Intrauterine IV	µg	Not
Zhidu et al. (2024)	PDLSCs DPSCs SCAPs SHEDs	50–300 µg 1 × 10^9^–2 × 10^10^ particles	Bone defect implantation Local injection	µg Particle number	Not
Zhu et al. (2025)	BM-MSCs UC-MSCs AD-MSCs	30–200 µg 1 × 10^9^–2 × 10^10^ particles	Topical hydrogel SC	µg Particle number	Yes

Abbreviations: MSC, mesenchymal stem cell; BM-MSCs, Bone Marrow-Derived Mesenchymal Stem Cells; UC-MSCs, Umbilical Cord-Derived Mesenchymal Stem Cells; AD-MSCs, Adipose Tissue-Derived Mesenchymal Stem Cells; WJ-MSCs, Wharton’s Jelly-Derived Mesenchymal Stem Cells; UCB-MSCs, Umbilical Cord Blood-Derived Mesenchymal Stem Cells; AF-MSCs, Amniotic Fluid-Derived Mesenchymal Stem Cells; ESC-MSCs, Embryonic Stem Cell-Derived Mesenchymal Stem Cells; IV, intravenous; IP, intraperitoneal; IT, intrathecal; IC, intracardiac; IN, intranasal; ICV, intracerebroventricular; IM, intramuscular; iPSC-MSCs, Induced Pluripotent Stem Cell-Derived Mesenchymal Stem Cells; PSC-MSCs, Pluripotent Stem Cell-Derived Mesenchymal Stem Cells; NSCs, Neural Stem Cells; EF-MSCs, Endometrial Fibroblast-Derived Mesenchymal Stem Cells; Dental MSCs, Dental Tissue-Derived Mesenchymal Stem Cells; DPSCs, Dental Pulp Stem Cells; SHEDs, Stem Cells from Human Exfoliated Deciduous Teeth; SCAPs, Stem Cells from Apical Papilla; PDLSCs, Periodontal Ligament Stem Cells; TMSCs, Tonsil-Derived Mesenchymal Stem Cells; AMSCs, Amniotic Membrane-Derived Mesenchymal Stem Cells; MenSCs, Menstrual Blood-Derived Mesenchymal Stem Cells; uMSCs, Uterine-Derived Mesenchymal Stem Cells; Placenta-MSCs, Placenta-Derived Mesenchymal Stem Cells; SC, subcutaneous.

Across the included meta-analyses, MSC-EV doses varied widely depending on disease model, administration route, and MSC source. The reported doses ranged from as low as 2 μg to as high as 700 μg of EV protein per injection, or from 1 × 10^5^ to 1 × 10^11^ particles per dose. Most studies administered EVs intravenously, although intranasal, intrathecal, subcutaneous, intrauterine, and local delivery via hydrogels or scaffolds were also frequently reported. A new supplementary table (Table 4) was created to summarize these dosing parameters, including dose units, routes, and whether dose-response relationships were investigated. Among the reviewed studies, approximately one-third conducted some form of dose-response assessment, with 100 μg per injection emerging as a commonly effective dose across multiple conditions, including spinal cord injury, ischemic stroke, and diabetic wound healing.

**FIGURE 4:**
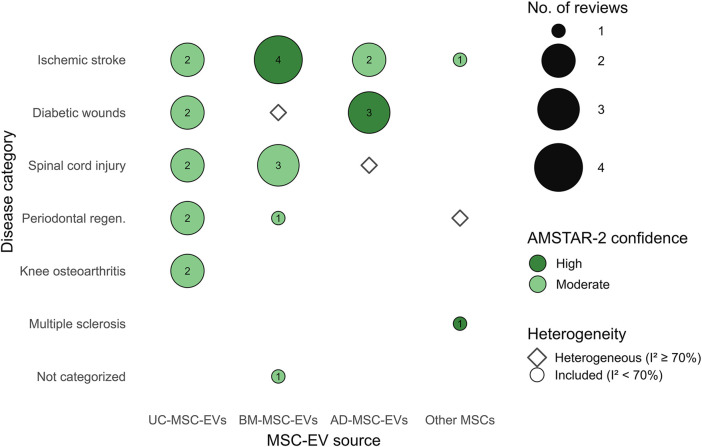
Bubble map of MSC-EV sources across disease categories, summarizing higher-quality meta-analyses. Filled bubbles indicate pairs supported by reviews with AMSTAR-2 High/Moderate confidence and I^2^ < 70%; bubble size encodes the number of such reviews. Hollow diamonds mark pairs reported with I^2^ ≥ 70% (promising but heterogeneous). Other MSCs aggregates less-frequent sources. Abbreviations: BM-MSC-EVs, bone marrow–derived; AD-MSC-EVs, adipose-derived; UC-MSC-EVs, umbilical cord–derived.

To integrate the evidence across sources and disease categories, we created a Bubble chart (Figure 4) mapping MSC-EV sources against disease models. This visualization includes only meta-analyses with AMSTAR-2 high or moderate confidence and I^2^ < 70%. Cells indicate the number of supporting meta-analyses, with darker shading representing stronger evidence. Hollow dots mark disease–source pairs where evidence exists but heterogeneity was high (I^2^ ≥ 70%). This figure highlights consistent support for BM-MSC-EVs in neurological diseases (stroke, SCI), AD-MSC-EVs in diabetic wound healing, and UC-MSC-EVs in musculoskeletal and periodontal regeneration.

### 3.2 Exosome source and therapeutic efficacy

The therapeutic efficacy of MSC-EVs varied notably depending on their cellular source (Table 5). Among the sources, BM-MSCs were the most extensively studied, with approximately 308 studies. These EVs demonstrated high effectiveness across multiple conditions, including ischemic stroke, SCI, acute kidney injury, and cardiovascular diseases. BMSC-EVs were particularly effective in reducing infarct size, improving neurological function scores, and promoting neuroregeneration.

**TABLE 5:** Comprehensive analysis of mesenchymal stem cell-derived extracellular vesicles sources and their therapeutic efficacy across diseases.

Stem cell source	Number of studies	Common disease targets	Key outcomes	Reported efficacy
Bone Marrow (BM-MSC)	308	Ischemic Stroke, IUA, TBI, Diabetic Wounds, Kidney Transplantation, Liver Diseases, NAFLD/NASH, SAH, Osteoporosis, DKD, SCI, Bone Injury, Osteosarcoma, AKI, CKD, POI, Asthma, Hemorrhagic Stroke, ALI/ARDS, Knee OA, MS, Cardiovascular Diseases, Periodontal Regeneration, Wound Healing, Liver Fibrosis	Reduced cerebral infarct volume (SMD -3.76), mNSS (SMD -2.11), SCr (MD -0.93 mg/dL), inflammation (TNF-α, IL-6, IL-1β; SMD -3.12), apoptosis (SMD -4.52), fibrosis, ALT, AST; improved AMH (SMD 5.39), BV/TV (WMD 14.07%), BBB score (WMD 3.47), wound closure (SMD 3.60), angiogenesis (SMD 4.64), EF (SMD 1.57)	High (less effective for kidney transplantation, acute/subacute ICH; best for revascularization)
Adipose Tissue (AD-MSC)	154	Ischemic Stroke, IUA, Diabetic Wounds, Sepsis, Kidney Transplantation, Liver Diseases, NAFLD/NASH, Osteoporosis, DKD, SCI, Bone Injury, Osteosarcoma, AKI, ED, CKD, Asthma, Hemorrhagic Stroke, ALI/ARDS, Knee OA, MS, Cardiovascular Diseases, TBI, POI, Wound Healing, Liver Fibrosis	Reduced inflammation (IL-6, TNF-α; SMD -2.30), cerebral infarct volume (SMD -3.76), SCr (MD -0.93 mg/dL), fibrosis, ALT, AST; improved wound closure (SMD 4.22), angiogenesis (SMD 4.64), AMH (SMD 5.39), BBB score (SMD -3.29), GFR	High (most effective for angiogenesis, wound closure; less effective for SCI, acute/subacute ICH)
Umbilical Cord (hUC-MSC)	119	Ischemic Stroke, IUA, Diabetic Wounds, Sepsis, Liver Diseases, NAFLD/NASH, SAH, Osteoporosis, DKD, SCI, Bone Injury, AKI, CKD, POI, Asthma, Hemorrhagic Stroke, ALI/ARDS, Knee OA, MS, Cardiovascular Diseases, TBI, Periodontal Regeneration, Wound Healing, Liver Fibrosis	Reduced inflammation (IL-6, TNF-α; SMD -2.30), cerebral infarct volume (SMD -3.76), SCr (MD -0.93 mg/dL), fibrosis, ALT, AST; improved AMH (SMD 5.39), wound closure (SMD 3.60), angiogenesis (SMD 4.64), BBB score (SMD -3.29), EF (SMD 1.57)	High (most effective for knee OA, periodontal regeneration)
Menstrual Blood (MenSC)	6	IUA, Diabetic Wounds, Liver Diseases, POI, Wound Healing	Reduced fibrosis, inflammation, ALT, AST; improved wound closure (SMD 3.60), angiogenesis, AMH, E2, pregnancy odds	High
Uterus (uMSC)	1	IUA	Reduced fibrosis; increased gland number	High
Synovial (SMSC)	6	Diabetic Wounds, Knee OA, Wound Healing	Reduced IL-1β, TNF-α, MMP-13; improved wound closure (SMD 3.60), angiogenesis, OARSI score, type II collagen, aggrecan, IL-10	High (superior for knee OA)
Decidua MSCs	2	Diabetic Wounds, Wound Healing	Reduced inflammation (IL-6; SMD -2.30); improved wound closure (SMD 3.16), angiogenesis (SMD 4.64), re-epithelialization (SMD 4.68)	High
Gingival MSCs	3	Diabetic Wounds, Type II Diabetic Wounds, Periodontal Regeneration	Reduced inflammation (IL-6; SMD -2.30); improved wound closure (SMD 3.16), angiogenesis (SMD 4.64), BV/TV (WMD 14.07%), CEJ-ABC (WMD -0.12 mm)	High
Amniotic (AMSC)	4	Liver Diseases, Wound Healing	Reduced ALT, AST, fibrosis; improved wound closure (SMD 3.60), angiogenesis, collagen deposition	High
Tonsil (TSC)	1	Liver Diseases	Reduced ALT, AST, fibrosis	High
Placental (hPMSC)	6	NAFLD/NASH, SCI, Asthma, Wound Healing	Reduced AST, ALT, inflammation, BALF IL-4; improved locomotion (BBB), neuro-regeneration, wound closure (SMD 3.60), angiogenesis	High
Urine-Derived (USC)	7	Osteoporosis, DKD, ED, CKD	Reduced SCr, BUN, inflammation; improved BMD, BV/TV, ICP/MAP, nNOS, eNOS, GFR	High
Wharton’s Jelly (hWJMSC)	2	SCI, AKI	Reduced inflammation, SCr, BUN, TNF-α; improved locomotion (BBB), neuro-regeneration, IL-10	High
Dental Pulp (DPSC)	5	SCI, Knee OA, Ischemic Stroke, Periodontal Regeneration	Reduced IL-1β, TNF-α, cerebral infarct volume; improved locomotion (BBB), OARSI score, BV/TV (WMD 14.07%), type II collagen, IL-10	High
Mouse Umbilical Cord (mUCMSC)	1	SCI	Reduced inflammation, GFAP; improved locomotion, neuro-regeneration	High
Kidney-Derived (KMSC)	2	AKI	Reduced SCr, BUN, TNF-α, apoptosis; increased IL-10	High
Human Liver Stem Cell (HLSC)	3	AKI, CKD	Reduced SCr, BUN, TNF-α, apoptosis; increased IL-10, GFR	High
Human Umbilical Cord Blood (hUCB-MSC)	8	DKD, CKD, ED	Reduced SCr, BUN, inflammation, fibrosis; improved IL-10, E-Cadherin, ICP/MAP, nNOS, eNOS, GFR	High
Muscle-Derived Stem Cells (MDSC)	1	ED	Improved ICP/MAP, nNOS, eNOS, smooth muscle/collagen ratio	High
Amniotic Fluid (AF-MSC)	11	CKD, POI, Knee OA	Reduced IL-1β, TNF-α, SCr, BUN; improved OARSI score, type II collagen, GFR, AMH, E2, pregnancy odds	High
Induced Pluripotent Stem Cell (iPSC-MSC)	6	POI, Asthma, Wound Healing	Reduced BALF IL-4; improved follicle count, AMH, E2, pregnancy odds, wound closure (SMD 3.60), angiogenesis	High
Clonal MSC (H-cMSC)	1	POI	Improved follicle count, AMH, E2, pregnancy odds	High
Periodontal Ligament (PDLSC)	9	MS, Periodontal Regeneration	Reduced inflammation (IL-17, IFN-γ, IL-1β), microglial activation; improved clinical score (SMD -2.17), BV/TV (WMD 14.07%), remyelination, Tregs	High (most effective for MS)
Neural Stem Cell (NSCEVs)	12	TBI, SCI	Reduced inflammation; improved mNSS (MD -2.0), BBB score (SMD 0.91), neuro-regeneration	High (early effect in SCI)
Dental Follicle Stem Cells (DFSCs)	2	Periodontal Regeneration	Improved BV/TV (WMD 14.07%), BMD (SMD 0.29); reduced CEJ-ABC (WMD -0.12 mm), Tb.Sp (SMD -0.08)	High (effective for bone regeneration)
Stem Cells from Human Exfoliated Deciduous Teeth (SHEDs)	2	Periodontal Regeneration	Improved BV/TV (WMD 14.07%), BMD (SMD 0.29); reduced CEJ-ABC (WMD -0.12 mm), Tb.Sp (SMD -0.08)	High (effective for bone regeneration)
Apical Papilla Stem Cells (SCAPs)	1	Periodontal Regeneration	Improved BV/TV (SMD 13.99), BMD (SMD 0.29); reduced CEJ-ABC (SMD -0.22), Tb.Sp (SMD -0.08)	High (effective for bone regeneration)
Hair Follicle MSCs	1	Wound Healing	Improved wound closure (SMD 3.60), angiogenesis, collagen deposition	High (superior for wound closure in diabetic models)
Oral Mucosa Lamina MSCs	3	Wound Healing	Improved wound closure (SMD 3.60), angiogenesis, collagen deposition	High
Orbicularis Oculi Muscle MSCs	1	Wound Healing	Improved wound closure (SMD 3.60), angiogenesis, collagen deposition	High

AD-MSCs, represented in about 154 studies, showed the highest efficacy in the treatment of diabetic wounds. These EVs promoted angiogenesis and accelerated wound closure, and also demonstrated consistent therapeutic benefits in models of liver fibrosis and chronic kidney disease.

hUC-MSCs, reported in around 119 studies, were most effective in models of knee osteoarthritis, periodontal tissue regeneration, and skin wound healing. hUC-MSC-EVs consistently reduced inflammation and improved functional outcomes across various disease models.

EVs derived from other MSC sources, such as menstrual blood, synovial tissue, and dental pulp, were less frequently studied but showed high therapeutic potential in specific conditions. For example, EVs from menstrual blood and synovial MSCs were effective in intrauterine adhesion and osteoarthritis, respectively, while periodontal ligament-derived EVs showed strong efficacy in models of multiple sclerosis and periodontal regeneration.

Notably, modified or engineered EVs—such as those loaded with specific microRNAs or preconditioned under hypoxic conditions—often outperformed their native counterparts. These engineered vesicles showed enhanced efficacy in models of stroke, SCI, and diabetic wounds. The method of EV delivery also influenced outcomes to some extent; while hydrogels and scaffold-based approaches were used in several studies, no delivery method demonstrated consistent superiority over direct injection.

### 3.3 Methodological quality and risk of bias

The methodological rigor of the included meta-analyses and their underlying primary studies revealed several key challenges (Table 6). Most reviews reported a moderate to high risk of bias, assessed using tools such as SYRCLE and CAMARADES. Common methodological shortcomings included unclear random sequence generation, lack of blinding of personnel and outcome assessors, and insufficient details regarding allocation concealment. Furthermore, publication bias was detected in several high-interest disease models—including stroke, SCI, and diabetic wounds—although many findings remained robust after trim-and-fill adjustments. Across the included reviews, the most frequent biases were inadequate or unclear random sequence generation, lack of blinding of investigators and outcome assessors, and insufficient allocation concealment. These issues were consistently reported in the majority of meta-analyses and represent systemic weaknesses in preclinical MSC-EV research.

**TABLE 6:** Comprehensive summary of risk of bias assessments in meta-analysis of mesenchymal stem cell-derived extracellular vesicles-based studies.

Authors, reference	Tool used	Overall RoB rating	Most common biases^a^
Aghayan et al. (2024)	Novel Tool	Unclear	Methodological heterogeneity, data extraction limitations
Bailey et al. (2022)	SYRCLE	Unclear	Unclear randomization, allocation concealment, blinding
Bernardi et al. (2025)	SYRCLE	Moderate-High	Allocation, blinding, random housing
Chen et al. (2024)	CAMARADES	Moderate	Sample size calculation, allocation concealment, blinding
Chen et al. (2023)	SYRCLE	High	Allocation concealment, performance bias, detection bias
Dai et al. (2025)	SYRCLE	Moderate	Allocation sequence, blinding, baseline similarity
Fang et al. (2023)	CAMARADES	Moderate	Sample size calculation, blinding, random outcome assessment
Fang et al. (2022)	SYRCLE	High	Selection bias (random allocation), attrition bias
Firouzabadi et al. (2024a)	SYRCLE	Moderate	Blinding, allocation concealment, random outcome assessment
Firouzabadi et al. (2024b)	SYRCLE	Low	Sequence generation, allocation concealment, blinding
Gunjan et al. (2024)	SYRCLE	Moderate	Blinding, allocation concealment
He et al. (2022)	CAMARADES	Moderate	Sample size calculation, blinded SAH induction
He et al. (2023)	SYRCLE	Moderate	Allocation concealment, blinding, random outcome assessment
Hickson et al. (2021)	SYRCLE	Moderate	Allocation concealment, blinding, random housing, outcome assessment
Himanshu et al. (2025)	SYRCLE	Moderate	Blinding, allocation concealment, random housing
Jabermoradi et al. (2025)	SYRCLE	Moderate	Allocation concealment, blinding, random housing, outcome assessment
Kirkham et al. (2022)	SYRCLE	Unclear	Blinding, allocation concealment, selective reporting, randomization
Liu et al. (2020a)	CAMARADES	Moderate	Sample size calculation, blinded model induction, blinded outcome assessment
Liu et al. (2024)	SYRCLE	Unclear	Blinding, random outcome assessment, allocation concealment
Lou et al. (2025)	Custom (9 criteria)	High-Moderate	Blinding, sample size calculation, follow-up duration
Lv et al. (2025)	SYRCLE	Moderate	Allocation concealment, blinding, randomization
Mou et al. (2025)	SYRCLE	Low	Minor issues in randomization, blinding
Nowak et al. (2022)	CAMARADES	Moderate	Randomization, blinded outcome assessment, conflict of interest statement
Shang et al. (2024)	SYRCLE	High	Unclear randomization, allocation concealment, limited blinding (25/40 studies)
Soltani et al. (2024)	SYRCLE	Unclear	Lack of randomization details, unclear allocation concealment, no blinding
Tieu et al. (2021)	SYRCLE	Moderate	Unclear randomization, allocation concealment, partial blinding of outcome assessors
Wang et al. (2024)	CAMARADES	High	Lack of blinding, no sample size calculation, unclear random housing
Wang et al. (2020)	SYRCLE	Moderate	Unclear randomization, allocation concealment, lack of blinding, variable assessment
Wang et al. (2025)	SYRCLE	Moderate	Unclear randomization (24/28 studies), allocation concealment, limited blinding
Wendt et al. (2018)	SYRCLE	Moderate	Unclear randomization, allocation concealment, limited blinding, variable EV reporting
Xu et al. (2024)	SYRCLE	Moderate	Unclear randomization (32/38 studies), allocation concealment, limited blinding
Xun et al. (2022)	SYRCLE	Unclear	Unclear randomization, allocation concealment, blinding, incomplete outcome reporting
Yang et al. (2023a)	SYRCLE	Moderate	Unclear randomization, allocation concealment, limited blinding, uneven study quality
Yang et al. (2022)	SYRCLE	Unclear	Unclear attrition bias, selective reporting (92% unclear), publication bias
Yang et al. (2023b)	SYRCLE	Moderate	Unclear randomization, allocation concealment, high heterogeneity (I^2^ = 94% for mNSS)
Ye et al. (2024)	SYRCLE	Unclear	Unclear randomization (29/30 studies), blinding, allocation concealment, publication bias
Yi and Wang (2021)	SYRCLE	Unclear	Unclear randomization, blinding, publication bias for BBB scores (Egger’s p = 0.00)
Yue et al. (2024)	SYRCLE	Unclear	Unclear randomization, allocation concealment, blinding, publication bias (Egger’s p = 0.000)
Zhang et al. (2016a)	SYRCLE	Unclear	Unclear randomization, allocation concealment, blinding, no publication bias
Zhang et al. (2016b)	SYRCLE	Unclear	Unclear randomization, allocation concealment, blinding, potential publication bias
Zhang et al. (2022)	SYRCLE	Moderate	Unclear randomization (22/24 studies), no allocation concealment, publication bias
Zhang et al. (2025)	CAMARADES	Moderate	Sample size calculation, unclear randomization, blinding
Zhou et al. (2023a)	SYRCLE, NIH	Unclear	Unclear randomization, limited blinding, publication bias for AMH
Zhou et al. (2025)	Cochrane	Unclear	Unclear randomization, allocation concealment, blinding, incomplete outcome data
Zhou et al. (2024)	SYRCLE	Unclear	Unclear randomization, allocation concealment, lack of blinding
Zhou et al. (2023b)	SYRCLE	Unclear	Unclear allocation concealment, blinding, high risk for random housing
Zhu et al. (2025)	SYRCLE	Unclear	Unclear randomization, allocation concealment, blinding, poor dose reporting

^a^ Common recurring issues across studies were unclear randomization procedures, lack of blinding, and poor allocation concealment.

**FIGURE 5:**
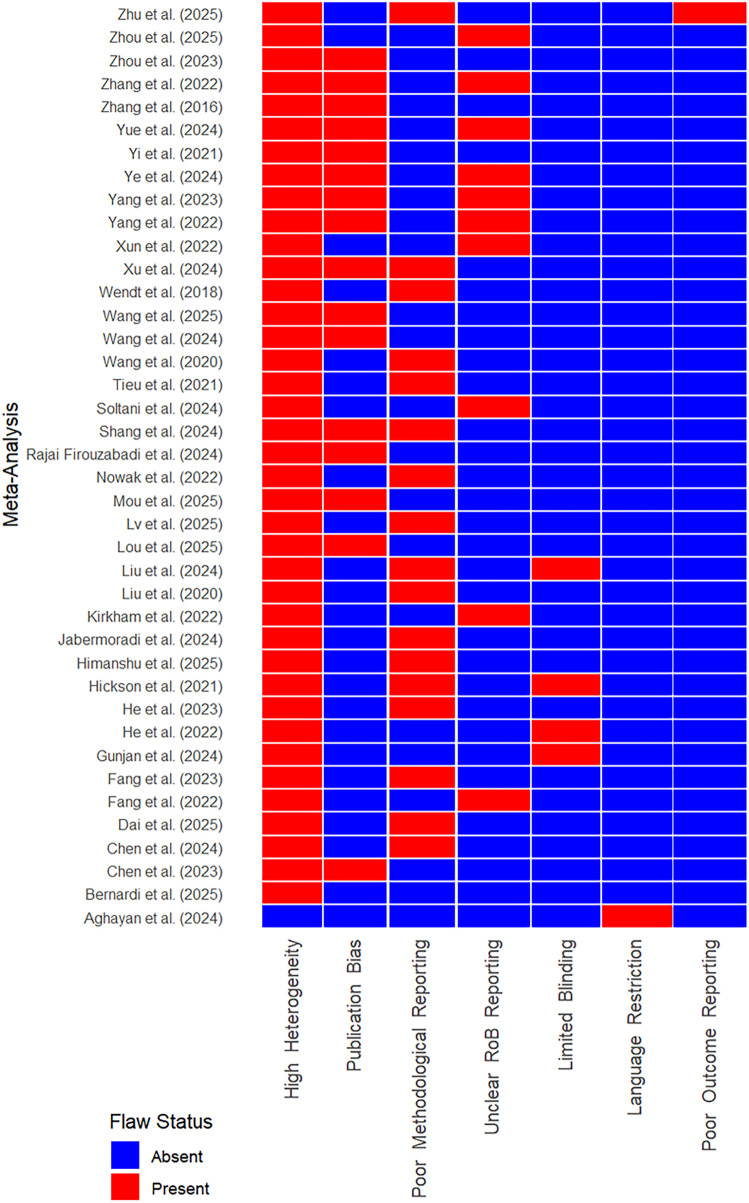
Distribution of critical flaws across meta-analysis of mesenchymal stem cell-derived extracellular vesicles-based studies in AMSTAR 2 assessments.

In terms of methodological quality, all included meta-analyses received a moderate AMSTAR 2 rating (Figure 5; Supplementary Table S2). This was primarily due to high heterogeneity (with I^2^ values ranging from 35% to 99%) and limited reporting of essential methodological components such as randomization procedures and blinding. Important methodological shortcomings were identified in several studies, particularly incomplete or unclear risk of bias assessments and lack of consideration for publication bias. Where AMSTAR-2 critical domains were rated ‘No,’ these reviews were classified as low or critically low confidence.

Heterogeneity was a significant concern across the dataset, with I^2^ values often exceeding 70%. This variability was largely attributed to differences in animal models, MSC sources, EV dosages, and delivery routes. Despite this, sensitivity and subgroup analyses frequently confirmed the robustness of results, suggesting that the therapeutic effects of MSC-EVs were consistent across different experimental conditions.

## 4 Discussion

### 4.1 Therapeutic efficacy and clinical implications

The review suggests that MSC-EVs exhibit high efficacy across multiple disease categories, including neurological, renal, wound healing, liver, musculoskeletal, respiratory, and reproductive disorders. Notably, MSC-EVs consistently reduced inflammation and apoptosis while promoting tissue regeneration, angiogenesis, and functional recovery. For instance, in ischemic stroke, MSC-EVs reduced cerebral infarct volume (SMD -3.76) and improved neurological scores (mNSS; SMD -2.11), with BMSC-EVs showing superior efficacy (Zhao et al., 2023). Similarly, in diabetic wounds, adipose-derived EVs (ADSC-EVs) accelerated wound closure (SMD 4.22) and enhanced angiogenesis (SMD 9.27), highlighting their potential in regenerative medicine (Soltani et al., 2024).

These findings align with the broader literature on MSC-EVs, which emphasizes their role as bioactive mediators carrying microRNAs, proteins, and lipids that modulate cellular processes. The high efficacy observed in conditions like SCI and traumatic brain injury, where MSC-EVs improved locomotor scores (BBB; WMD 3.47) and cognitive outcomes (mNSS; SMD -4.48), underscores their neuroprotective and regenerative capabilities (Chen et al., 2024; Ye et al., 2024). The ability of MSC-EVs to outperform conditioned medium in acute kidney injury (Liu C. et al., 2020; Zhang G. et al., 2016) and to match or exceed MSC-based therapies in subarachnoid hemorrhage (He et al., 2022) further supports their therapeutic advantage, likely due to their stability, low immunogenicity, and ability to cross biological barriers.

The clinical implications are significant. MSC-EVs offer a cell-free therapeutic approach that circumvents challenges associated with MSC transplantation, such as immune rejection and tumorigenic risks. Their efficacy in diverse preclinical models suggests potential for broad clinical applications, particularly in conditions with high unmet needs, such as stroke, SCI, and diabetic complications. However, the variability in efficacy across diseases highlights the need for disease-specific optimization of EV sources, dosing, and delivery methods.

### 4.2 Exosome source and optimization

The review reveals that exosome source significantly influences therapeutic outcomes. AD-MSC-EVs excelled in wound healing, particularly diabetic wounds, where they promoted angiogenesis and collagen deposition, while BM-MSC-EVs demonstrated superior effects in neurological models. hUC-MSCs showed superior efficacy in knee osteoarthritis and periodontal regeneration, possibly due to their high proliferative capacity and immunomodulatory properties.

Emerging sources, such as periodontal ligament (PDLSCs) for multiple sclerosis (Xun et al., 2022) and menstrual blood (MenSCs) for intrauterine adhesion (Chen et al., 2023), demonstrated high efficacy despite fewer studies, suggesting untapped potential. Modified EVs, such as miRNA-loaded or hypoxia-pretreated EVs, consistently outperformed native EVs, as seen in SCI (Hu et al., 2021; Liu W. et al., 2020; Yang et al., 2024) and stroke (Li et al., 2023; Song et al., 2024), where engineered EVs enhanced functional recovery by targeting specific pathways. These findings align with recent studies emphasizing the role of EV cargo engineering in enhancing therapeutic specificity.

Delivery methods also influenced outcomes. Intravenous and intrathecal routes were most common, with intrathecal administration showing superior efficacy in SCI. Hydrogels and scaffolds improved outcomes in some contexts, but their benefit was not universal, as seen in diabetic wounds where non-hydrogel methods were equally effective (Bailey et al., 2022; Chen et al., 2025). These observations underscore the need for tailored delivery strategies based on disease pathophysiology and target tissue.

The administration route is another determinant of therapeutic outcomes. While intravenous delivery remains the most frequently used method, it may not be optimal for all disease contexts. For CNS conditions, intrathecal and intranasal delivery were more effective in bypassing the blood–brain barrier and enhancing neuroprotective outcomes. For local pathologies, such as wounds and periodontal disease, local injection and hydrogel-mediated delivery improved retention and tissue-specific effects. These observations underscore the need for future preclinical and clinical studies to systematically evaluate route-dependent biodistribution and efficacy of MSC-EVs.

This crosswalk illustrates the concentration of higher-quality evidence, showing clear clusters of BM-MSC-EVs with neurological models, AD-MSC-EVs with wound healing, and UC-MSC-EVs with musculoskeletal and periodontal regeneration. These patterns emphasize the importance of tailoring MSC-EV source selection to disease context.

### 4.3 Considerations on MSC-EV dose optimization

One critical but under-addressed variable in MSC-EV therapy is dosing strategy. Our umbrella review found substantial variability in reported doses, with most studies using a fixed dose (often 100 μg) without justification or titration. While several studies—such as those on SCI, stroke, and reproductive models—performed subgroup or network meta-analyses to examine dose-response relationships, the overall evidence remains fragmented and underpowered. In some cases, 100–200 μg was reported as optimal for neuroprotection or tissue regeneration, yet other studies used much higher doses (up to 700 μg) or particle-based quantifications (1 × 10^9^ to 10^11^ particles).

The lack of standardized dosing metrics (mass vs. particle count), inconsistent reporting of EV characterization, and variable injection regimens further complicate cross-study comparisons. Notably, some studies administered EVs via specialized delivery systems, which could enhance local bioavailability and reduce systemic loss. However, head-to-head comparisons across these delivery platforms remain limited.

To support clinical translation, future preclinical trials should incorporate formal dose-response analyses, adopt standardized reporting in line with MISEV2023 guidelines, and evaluate pharmacokinetics and tissue distribution in parallel with efficacy outcomes (Su et al., 2025).

### 4.4 Mechanisms of action

The therapeutic effects of MSC-EVs are mediated through multiple mechanisms, including anti-inflammatory, anti-apoptotic, and regenerative pathways (Liao et al., 2022). The consistent reduction in proinflammatory cytokines and upregulation of IL-10 across diseases like asthma, sepsis, and liver fibrosis highlight their immunomodulatory role. In neurological disorders, MSC-EVs reduced neuronal apoptosis and promoted neurogenesis and axonal regeneration, contributing to functional recovery (Dabrowska et al., 2020). In wound healing, enhanced angiogenesis and collagen deposition were driven by EV-mediated delivery of growth factors and microRNAs (Pulido-Escribano et al., 2023).

These mechanisms are consistent with the literature, which attributes MSC-EV efficacy to their cargo of bioactive molecules, including miRNAs, proteins, and lipids. The ability of MSC-EVs to modulate multiple pathways simultaneously explains their broad efficacy but also complicates efforts to pinpoint specific mechanisms for each disease (Tsuji et al., 2020). Future studies should leverage omics technologies to elucidate disease-specific EV cargos and their targets, facilitating precision medicine approaches.

### 4.5 Methodological quality and limitations

A major limitation across the evidence base is the prevalence of randomization bias, lack of blinding, and inadequate allocation concealment, as summarized in Table 6. These issues undermine internal validity and may inflate reported effect sizes. The review identified significant methodological challenges that temper the interpretation of findings. Most meta-analyses reported moderate to high risk of bias, primarily due to unclear randomization, lack of blinding, and inadequate allocation concealment in primary studies. The SYRCLE and CAMARADES tools highlighted these issues, with only a few studies achieving low risk across all domains. High heterogeneity (I^2^ often >70%) was another concern, driven by variations in animal models, EV sources, doses, and administration protocols. While sensitivity analyses and trim-and-fill adjustments often confirmed robust findings, publication bias was evident in conditions like stroke and SCI, suggesting a potential overestimation of effect sizes. Although some outcomes showed very large effect sizes, they were accompanied by high heterogeneity (I^2^ ≥ 70%). In this umbrella review, we did not exclude these results but reclassified them as Promising but heterogeneous to preserve comprehensiveness while reflecting their limited certainty.

The AMSTAR 2 assessments rated all meta-analyses as moderate quality, reflecting limitations in reporting randomization, blinding, and publication bias assessments. The lack of standardized EV characterization further complicates comparisons across studies. These methodological issues align with broader challenges in preclinical research, where poor reporting and experimental design can undermine reproducibility (Simon-Tillaux et al., 2022).

The umbrella review itself has limitations. The restriction to English-language studies may have excluded relevant non-English meta-analyses (Wang et al., 2015). The reliance on reported data from included meta-analyses meant that incomplete or inconsistent reporting could affect our synthesis. Additionally, the diversity of diseases and outcomes precluded a formal meta-analysis of effect sizes, limiting our ability to quantify overall efficacy.

### 4.6 Limitations and considerations

Because umbrella reviews rely on published meta-analyses, we cannot exclude or re-pool individual primary studies. Instead, we downgraded evidence strength for outcomes with I^2^ ≥ 70% to Promising but heterogeneous. This ensures transparency while retaining the comprehensive scope of the umbrella review.

Several limitations must be considered when interpreting the findings of this umbrella review. Study quality was a notable concern, as poor reporting of critical methodological aspects such as randomization, blinding, and allocation concealment limited the reliability of some conclusions. Many primary studies scored between 3 and 7 on the SYRCLE scale, reflecting low to moderate methodological quality.

Several included reviews were of low or critically low confidence according to AMSTAR-2, and while retained for completeness, sensitivity summaries excluding these reviews are presented to indicate robustness of conclusions.

Future preclinical MSC-EV studies should implement rigorous randomization and blinding, with transparent allocation concealment, in line with ARRIVE reporting standards, to improve the reliability of pooled evidence.

Publication bias was evident in numerous conditions, including stroke, SCI, and post-operative ileus, as indicated by asymmetrical funnel plots and significant Egger’s or Begg’s test results. However, subsequent trim-and-fill analyses often confirmed the stability of the observed effects, lending credibility to the synthesized outcomes.

Another issue was the variability in exosome characterization. Some studies did not include essential quality control data, such as electron microscopy images or expression analysis of EV surface markers, which may affect the comparability and reproducibility of MSC-EV therapies.

Lastly, translational challenges remain. While MSC-EVs demonstrated high efficacy across a range of preclinical disease models, differences in dosing regimens, timing of administration, and delivery strategies must be standardized to advance these findings toward clinical application.

### 4.7 Future directions

Several key priorities have emerged to guide future research on MSC-EVs, with the goal of enhancing scientific rigor and accelerating clinical translation. First and foremost, there is a critical need for standardization. Uniform protocols for EV isolation, characterization, and dosing must be developed and widely adopted to ensure reproducibility and comparability across studies. In this context, strict adherence to the MISEV2023 (Minimal Information for Studies of Extracellular Vesicles) guidelines should be considered essential (Welsh et al., 2024).

In addition, mechanistic studies should be expanded using advanced omics technologies—such as proteomics, transcriptomics, and metabolomics—alongside bioinformatics tools, to elucidate disease-specific EV cargos and their molecular targets. Such insights will support the development of more tailored and effective therapeutic strategies. Optimization of MSC-EV therapies is another important area of focus. This includes exploring novel and less-studied EV sources, such as PDLSCs and MenSCs, as well as employing bioengineering strategies like microRNA loading or surface modification to enhance therapeutic potency.

Across the included meta-analyses, the most commonly reported methods were hypoxic preconditioning, miRNA-engineering, cytokine/growth factor priming, and scaffold-based conditioning. These preconditioning approaches were consistently associated with improved therapeutic efficacy, including enhanced angiogenesis, neuroprotection, and anti-inflammatory effects. For example, hypoxia-enhanced EVs showed superior functional outcomes in spinal cord injury models, while miRNA-modified EVs demonstrated targeted regulation of inflammatory and regenerative pathways (Jiang et al., 2025). Scaffold incorporation also supported sustained EV release and localized tissue repair (Leung et al., 2022). These findings suggest that preconditioning may be a key determinant of EV potency, and future research should prioritize standardized evaluation of these strategies (Liu et al., 2025).

For clinical translation, the field must now progress toward conducting early-phase clinical trials (Phase I/II) to assess the safety, tolerability, and efficacy of MSC-EVs in human subjects. Priority should be given to high-impact conditions where preclinical data already show strong therapeutic potential, such as ischemic stroke and diabetic wounds. Alongside these translational efforts, improving methodological rigor in preclinical studies is crucial. This involves proper implementation of randomization, blinding, and allocation concealment, with transparent reporting practices aligned with the ARRIVE (Animal Research: Reporting of *In Vivo* Experiments) guidelines.

Finally, addressing publication bias remains a vital consideration. The use of prospective study registration and open-access data platforms can help ensure that both positive and negative results are reported, thereby strengthening the integrity of the evidence base. By tackling these research priorities, the field can move toward more reliable, effective, and clinically applicable MSC-EV therapies.

## 5 Conclusion

MSC-EVs demonstrate remarkable therapeutic potential across diverse preclinical models, with high efficacy in reducing inflammation, apoptosis, and tissue damage while promoting regeneration and functional recovery. BM-, adipose-, and umbilical cord-derived EVs are particularly promising, with modified EVs offering enhanced benefits. Despite methodological limitations, the consistency of positive outcomes supports MSC-EVs as a viable therapeutic strategy. However, current studies are limited by small sample sizes, heterogeneous isolation and characterization methods, and variable outcome measures, which hinder comparability and reproducibility. Future studies should prioritize standardized protocols, robust mechanistic investigations, and rigorous experimental design to address these shortcomings. Addressing standardization, mechanistic understanding, and study quality will be critical to translating these findings into clinical practice, potentially revolutionizing treatment for a wide range of diseases.
