# Supplementary material for: Umbrella review of mesenchymal stem cell-derived extracellular vesicles in preclinical models: therapeutic efficacy across diverse conditions
Source: Front Cell Dev Biol. 2025 Oct 13;13:1655623. doi: 10.3389/fcell.2025.1655623 (PMC12554768; doi:10.3389/fcell.2025.1655623)
Supplement: Supplementary file 1 [file Table1.docx]

Table S1. Effectiveness of mesenchymal stem cell-derived extracellular vesicles across outcomes for various diseases

| Disease/Condition | Inflammation | Apoptosis | Function Score | Histology |
| --- | --- | --- | --- | --- |
| Acute Kidney Injury | High (reduced TNF-α, IL-6, IL-1β; Zhang 2016) | High (reduced apoptosis; Zhang 2016) | High (SCr: MD 0.93, 95% CI 0.67–1.20; Zhang 2016) | High (improved renal repair; Zhang 2016) |
| Asthma | High (reduced TNF-α, IL-6, IL-1β) | Not reported | High (improved lung function) | High (reduced lung injury) |
| Bone Injury | Moderate (reduced inflammation) | Not reported | Not reported | High (increased BV/TV, NBF) |
| Cardiovascular Diseases | High (reduced TNF-α, IL-6; Wendt 2018, Zhang 2016) | High (reduced caspase activity; Wendt 2018, Zhang 2016) | High (EF: SMD 1.57, 95% CI 0.86–1.26; Zhang 2016) | High (reduced infarct, increased angiogenesis; Wendt 2018, Zhang 2016) |
| Chronic Kidney Disease | High (reduced TNF-α, IL-6, IL-1β) | High (reduced apoptosis) | High (improved GFR, SCr) | High (improved renal histology) |
| Diabetic Kidney Disease | High (reduced TNF-α, IL-6, IL-1β) | Moderate (reduced apoptosis) | High (reduced SCr, BUN) | High (reduced fibrosis) |
| Diabetic Wounds | High (reduced IL-6, TNF-α, IL-1β; Soltani 2024, Yue 2024) | Moderate (reduced apoptosis; Soltani 2024) | High (closure: SMD 4.22, 95% CI 3.07–5.36; Soltani 2024) | High (increased collagen, angiogenesis; Soltani 2024, Yue 2024) |
| Erectile Dysfunction | Moderate (reduced inflammation) | Moderate (reduced apoptosis) | High (improved ICP/MAP) | High (improved tissue repair) |
| Hemorrhagic Stroke | High (reduced TNF-α, IL-6, IL-1β) | High (reduced neuronal apoptosis) | High (SAH: SMD -3.49, 95% CI -4.23 to -2.75; ICH: SMD 2.38) | High (increased angiogenesis, neurogenesis) |
| Intrauterine Adhesion | High (reduced IL-1β, IL-6, TNF-α; increased IL-10; Zhou 2023) | Moderate (reduced apoptosis; Zhou 2023) | High (endometrial thickness: WMD 132.36, 95% CI 118.99–145.74; Zhou 2023) | High (reduced fibrosis, increased CK19; Zhou 2023) |
| Ischemic Stroke | High (reduced TNF-α: SMD -2.60; IL-1β: SMD -2.57; Zhang 2022, 2025) | High (reduced caspase-3: SMD -5.40; Zhang 2022, 2025) | High (mNSS: SMD -2.11, 95% CI -2.51 to -1.70; Zhang 2022, 2025) | High (increased neurovascular repair; Zhang 2022, Xu 2024, Zhang 2025) |
| Kidney Transplantation | Not reported | Not reported | Low (MSC-EVs ineffective) | Not reported |
| Knee Osteoarthritis | High (reduced IL-1β, IL-6, TNF-α, MMP-13) | High (reduced chondrocyte apoptosis) | High (OARSI: SMD -2.97, 95% CI -3.62 to -2.31) | High (increased collagen II, cartilage repair) |
| Liver Diseases | High (reduced TNF-α, IL-6, IL-1β) | Not reported | High (improved ALT, AST) | High (reduced fibrosis) |
| Liver Fibrosis | Moderate (reduced TNF-α, IL-6; Zhou 2025) | Moderate (reduced apoptosis; Zhou 2025) | High (ALT: SMD -3.14, 95% CI -3.62 to -2.66; Zhou 2025) | High (reduced collagen: SMD -2.92, 95% CI -4.76 to -1.08; Zhou 2025) |
| Multiple Sclerosis | High (reduced IL-17, IFN-γ, IL-1β; Xun 2022) | High (reduced caspase-3, Bax; Xun 2022) | High (clinical score: SMD -2.17, 95% CI -3.99 to -0.34; Xun 2022) | High (increased remyelination) |
| NAFLD/NASH | High (reduced TNF-α, IL-6, IL-1β) | Not reported | High (improved ALT, AST) | High (reduced steatosis) |
| Osteoporosis | Not reported | High (reduced bone cell apoptosis) | Not reported | High (improved BMD) |
| Osteosarcoma | Not reported | Not reported | High (reduced tumor volume) | High (improved tissue architecture) |
| Periodontal Regeneration | Moderate (reduced inflammation; Zhou 2023, 2025) | Moderate (reduced apoptosis; Zhou 2023, 2025) | Not reported | High (BV/TV: WMD 14.07, 95% CI 6.73–21.41; Zhou 2025) |
| Premature Ovarian Insufficiency | High (reduced IL-1β, IL-6, TNF-α; increased IL-10; Zhou 2023) | High (reduced granulosa cell apoptosis; Zhou 2023) | High (AMH: SMD 5.39, 95% CI 3.43–7.36; Zhou 2023) | High (increased follicle numbers; Zhou 2023) |
| Respiratory Diseases | High (reduced TNF-α, IL-6, IL-1β; Wang 2020) | Moderate (reduced apoptosis; Wang 2020) | High (survival: OR 6.45, 95% CI 2.78–14.97; Wang 2020) | High (reduced lung injury: SMD -4.02; Wang 2020) |
| Sepsis | High (reduced TNF-α, IL-6, IL-1β; Aghayan 2024) | Moderate (reduced apoptosis) | High (improved survival; Aghayan 2024) | Moderate (reduced tissue damage) |
| Spinal Cord Injury | High (reduced TNF-α: SMD -3.12; increased IL-10; Ye 2024, Yang 2022) | High (reduced apoptosis: SMD -4.52; Ye 2024, Yang 2022) | High (BBB: WMD 3.47, 95% CI 3.31–3.63; Ye 2024, Shang 2024) | High (increased NeuN: SMD 3.54; reduced GFAP; Ye 2024) |
| Subarachnoid Hemorrhage | High (reduced TNF-α, IL-6, IL-1β; He 2022) | Not reported | High (improved neurobehavior; He 2022) | Moderate (improved tissue repair) |
| Traumatic Brain Injury | High (reduced TNF-α, IL-1β, IL-6; Yang 2023) | High (reduced neuronal apoptosis; Yang 2023) | High (mNSS: SMD -4.48, 95% CI -6.12 to -2.84; Yang 2023) | High (increased neurogenesis; Yang 2023) |
| Type II Diabetic Wounds | High (reduced IL-6: SMD -2.30; increased IL-10: SMD 2.04; Yue 2024) | Moderate (reduced apoptosis; Yue 2024) | Not reported | High (angiogenesis: SMD 4.64; collagen: SMD 4.01; Yue 2024) |
| Wound Healing/Skin Regeneration | High (reduced IL-6, TNF-α, IL-1β; Zhu 2025) | High (reduced apoptosis; Zhu 2025) | High (closure: SMD 3.60, 95% CI 3.23–3.96; Zhu 2025) | High (increased angiogenesis, collagen; Zhu 2025) |

Abbreviations: SCr = Serum creatinine; EF = Ejection fraction; FS = Fractional shortening; EDV = End-diastolic volume; ESV = End-systolic volume; mNSS = Modified Neurological Severity Score; BBB = Basso, Beattie, Bresnahan; OARSI = Osteoarthritis Research Society International score; BV/TV = Bone volume/total volume; CEJ-ABC = Cementoenamel junction-alveolar bone crest; AMH = Anti-Müllerian hormone; SMD = Standardized mean difference; WMD = Weighted mean difference; MD = Mean difference; CI = Confidence interval; TNF-α = Tumor necrosis factor-alpha; IL = Interleukin; GFAP = Glial fibrillary acidic protein; NeuN = Neuronal nuclei; CK19 = Cytokeratin 19.

Table S2. Distribution of critical flaws across meta-analysis of mesenchymal stem cell-derived extracellular vesicles-based studies in AMSTAR 2 assessments

| Meta-Analysis | AMSTAR 2 Rating | Critical Flaws | Overall Confidence |
| --- | --- | --- | --- |
| Aghayan et al. (2024) | Moderate | Q2: Language restriction | Moderate |
| Bernardi et al. (2025) | Moderate | Q9: Limited RoB reporting | Moderate |
| Chen et al. (2023) | Moderate | Q9: High heterogeneity, publication bias | Moderate |
| Chen et al. (2024) | Moderate | Q9: Poor randomization/blinding reporting | Moderate |
| Dai et al. (2025) | Moderate | Q9: High heterogeneity, poor methodological reporting | Moderate |
| Fang et al. (2022) | Moderate | Q9: High heterogeneity, unclear RoB | Moderate |
| Fang et al. (2023) | Moderate | Q9: High heterogeneity, incomplete reporting | Moderate |
| Gunjan et al. (2024) | Moderate | Q9: High heterogeneity, limited blinding | Moderate |
| He et al. (2022) | Moderate | Q9: High heterogeneity, limited blinding | Moderate |
| He et al. (2023) | Moderate | Q9: High heterogeneity, poor methodological reporting | Moderate |
| Hickson et al. (2021) | Moderate | Q9: High heterogeneity, limited blinding/reporting | Moderate |
| Himanshu et al. (2025) | Moderate | Q9: High heterogeneity, limited methodological reporting | Moderate |
| Jabermoradi et al. (2024) | Moderate | Q9: High heterogeneity, poor randomization/blinding reporting | Moderate |
| Kirkham et al. (2022) | Moderate | Q9: High heterogeneity, unclear RoB reporting | Moderate |
| Liu et al. (2020) | Moderate | Q9: High heterogeneity, limited methodological reporting | Moderate |
| Liu et al. (2024) | Moderate | Q9: High heterogeneity, limited blinding/reporting | Moderate |
| Lou et al. (2025) | Moderate | Q9: High heterogeneity, publication bias | Moderate |
| Lv et al. (2025) | Moderate | Q9: High heterogeneity, limited methodological reporting | Moderate |
| Mou et al. (2025) | Moderate | Q9: High heterogeneity, potential publication bias | Moderate |
| Nowak et al. (2022) | Moderate | Q9: High heterogeneity, limited methodological reporting | Moderate |
| Rajai Firouzabadi et al. (2024, Asthma) | Moderate | Q9: High heterogeneity, publication bias | Moderate |
| Rajai Firouzabadi et al. (2024, POI) | Moderate | Q9: High heterogeneity, publication bias | Moderate |
| Soltani et al. (2024) | Moderate | Q9: High heterogeneity (I² = 87.97% for wound closure), unclear RoB reporting (randomization, blinding, allocation concealment) | Moderate |
| Shang et al. (2024) | Moderate | Q9: High heterogeneity (funnel plot asymmetry, potential publication bias), poor methodological reporting (randomization, blinding) | Moderate |
| Tieu et al. (2021) | Moderate | Q9: High heterogeneity (I² = 77–95%), limited methodological reporting (randomization, blinding, allocation concealment) | Moderate |
| Wang et al. (2020) | Moderate | Q9: High heterogeneity (I² = 67–73%), limited methodological reporting (no explicit RoB tool, blinding unclear) | Moderate |
| Wang et al. (2024) | Moderate | Q9: High heterogeneity (I² = 92% for chronic ICH), publication bias (Egger’s test p=0.001 for SAH), limited blinding reporting | Moderate |
| Wang et al. (2025) | Moderate | Q9: High heterogeneity (I² = 58–81% for OARSI score, TNF-α), potential publication bias (asymmetry in TNF-α funnel plot) | Moderate |
| Wendt et al. (2018) | Moderate | Q9: High heterogeneity (not quantified), limited methodological reporting (EV characterization, blinding) | Moderate |
| Xu et al. (2024) | Moderate | Q9: High heterogeneity (I² not fully reported), publication bias (Begg’s/Egger’s p<0.001 for infarct volume, mNSS), limited methodological reporting | Moderate |
| Xun et al. (2022) | Moderate | Q9: High heterogeneity (I² = 84%), unclear RoB reporting (randomization, blinding, allocation concealment) | Moderate |
| Yang et al. (2022) | Moderate | Q9: High heterogeneity (funnel plot asymmetry, publication bias via Egger’s test), unclear RoB reporting (blinding, randomization, allocation concealment) | Moderate |
| Yang et al. (2023) | Moderate | Q9: High heterogeneity (not quantified, but uneven study quality), limited methodological reporting (randomization, blinding), no publication bias (Egger’s p=0.0986 for mNSS) | Moderate |
| Yang et al. (2023, 2) | Moderate | Q9: High heterogeneity (I² = 94% for mNSS, 76% for Foot Fault Test), unclear RoB reporting (randomization, allocation concealment, blinding), potential publication bias | Moderate |
| Ye et al. (2024) | Moderate | Q9: High heterogeneity (I² = 80.9% for BBB, 75.3% for TNF-α), unclear RoB reporting (randomization, blinding, allocation concealment), publication bias via funnel plot asymmetry | Moderate |
| Yue et al. (2024) | Moderate | Q9: Moderate to high heterogeneity (I² = 39% for wound closure, 72% for angiogenesis, 56% for collagen deposition), unclear RoB reporting (randomization, allocation concealment, blinding), significant publication bias (Egger’s test, p=0.000) | Moderate |
| Yi et al. (2021) | Moderate | Q9: High heterogeneity (I² = 78% for BBB, 73% for last measurement), unclear randomization and blinding, publication bias for BBB scores at last measurement (Egger’s test, p=0.00) | Moderate |
| Zhang et al. (2016) | Moderate | Q9: High heterogeneity (I² = 96% for SCr), unclear RoB reporting (randomization, blinding, allocation concealment), no publication bias detected | Moderate |
| Zhang et al. (2016, 2) | Moderate | Q9: High heterogeneity (I² = 94% for EF, 86.2% for FS, 94% for EDV, 90.4% for ESV), unclear RoB reporting (randomization, blinding, allocation concealment), potential publication bias due to positive outcome emphasis | Moderate |
| Zhang et al. (2022) | Moderate | Q9: Moderate heterogeneity (I² = 43% for infarct volume, 48% for mNSS, 35–44% for other outcomes), unclear RoB reporting (randomization, blinding, allocation concealment), publication bias via funnel plot asymmetry | Moderate |
| Zhou et al. (2023) | Moderate | Q9: Moderate to high heterogeneity (I² = 54.09% for endometrial thickness, 93.4% for glands in IUA), unclear RoB reporting (randomization, blinding, no sample size calculation), publication bias for AMH (Egger’s p=0.002) but not endometrial thickness (p=0.11) | Moderate |
| Zhou et al. (2025) | Moderate | Q9: High heterogeneity (I² = 70–91% for single administration, 0–89% for MSC-exos-drugs), unclear RoB reporting (randomization, allocation concealment, blinding), no publication bias due to limited studies | Moderate |
| Zhou et al. (2025, 3) | Moderate | Q9: High heterogeneity (I² = 99% for BV/TV, 36% for CEJ-ABC), unclear RoB reporting (allocation concealment, blinding, random housing), no publication bias (Egger’s p=0.43 for CEJ-ABC, p=0.55 for BV/TV) | Moderate |
| Zhu et al. (2025) | Moderate | Q9: High heterogeneity (I² = 83% overall, 85% diabetic, 82% non-diabetic), unclear RoB reporting (randomization, blinding, sample size, dose), poor outcome reporting, no publication bias reported | Moderate |
